# A Systems Approach Identifies Essential FOXO3 Functions at Key Steps of Terminal Erythropoiesis

**DOI:** 10.1371/journal.pgen.1005526

**Published:** 2015-10-09

**Authors:** Raymond Liang, Genís Campreciós, Yan Kou, Kathleen McGrath, Roberta Nowak, Seana Catherman, Carolina L. Bigarella, Pauline Rimmelé, Xin Zhang, Merlin Nithya Gnanapragasam, James J. Bieker, Dmitri Papatsenko, Avi Ma’ayan, Emery Bresnick, Velia Fowler, James Palis, Saghi Ghaffari

**Affiliations:** 1 Department of Developmental & Regenerative Biology, Icahn School of Medicine at Mount Sinai, New York, New York, United States of America; 2 Developmental and Stem Cell Biology Multidisciplinary Training Area, Icahn School of Medicine at Mount Sinai, New York, New York, United States of America; 3 Department of Pharmacology and Systems Therapeutics, Icahn School of Medicine at Mount Sinai, New York, New York, United States of America; 4 Department of Pediatrics, Center for Pediatric Biomedical Research,University of Rochester Medical Center, Rochester, New York, United States of America; 5 Department of Cell and Molecular Biology, Scripps Research Institute, La Jolla, California, United States of America; 6 Black Family Stem Cell Institute, Icahn School of Medicine at Mount Sinai, New York, New York, United States of America; 7 Tisch Cancer Institute, Icahn School of Medicine at Mount Sinai, New York, New York, United States of America; 8 Department of Cell and Regenerative Biology, UW-Madison Blood Research Program, University of Wisconsin, Madison, Wisconsin, United States of America; 9 Department of Medicine, Division of Hematology, Oncology, Icahn School of Medicine at Mount Sinai, New York, New York, United States of America; Centre for Cancer Biology, SA Pathology, AUSTRALIA

## Abstract

Circulating red blood cells (RBCs) are essential for tissue oxygenation and homeostasis. Defective terminal erythropoiesis contributes to decreased generation of RBCs in many disorders. Specifically, ineffective nuclear expulsion (enucleation) during terminal maturation is an obstacle to therapeutic RBC production *in vitro*. To obtain mechanistic insights into terminal erythropoiesis we focused on FOXO3, a transcription factor implicated in erythroid disorders. Using an integrated computational and experimental systems biology approach, we show that FOXO3 is essential for the correct temporal gene expression during terminal erythropoiesis. We demonstrate that the FOXO3-dependent genetic network has critical physiological functions at key steps of terminal erythropoiesis including enucleation and mitochondrial clearance processes. FOXO3 loss deregulated transcription of genes implicated in cell polarity, nucleosome assembly and DNA packaging-related processes and compromised erythroid enucleation. Using high-resolution confocal microscopy and imaging flow cytometry we show that cell polarization is impaired leading to multilobulated *Foxo3* ^*-/-*^ erythroblasts defective in nuclear expulsion. Ectopic FOXO3 expression rescued *Foxo3* ^*-/-*^ erythroblast enucleation-related gene transcription, enucleation defects and terminal maturation. Remarkably, FOXO3 ectopic expression increased wild type erythroblast maturation and enucleation suggesting that enhancing FOXO3 activity may improve RBCs production. Altogether these studies uncover FOXO3 as a novel regulator of erythroblast enucleation and terminal maturation suggesting FOXO3 modulation might be therapeutic in disorders with defective erythroid maturation.

## Author Summary

Red blood cells (RBCs) are highly specialized cells that transport oxygen throughout the body and are essential for survival. However, RBCs have a limited lifespan and need to be replenished continuously by stem cells in the bone marrow. Mammalian RBCs are unique in that in order to fully mature they exclude their nucleus and other organelles. Mechanisms involved in these processes are not well understood at the molecular level. Defects in any of the these processes may lead to red blood cell defects, a decreased capacity to transport oxygen and/or a block in red blood cell production in vitro. Therefore, understanding how these processes are regulated at the molecular level can lead to promising new therapies for red blood cell defects and improved methods of generating red blood cells in a dish. Here, using an integrated computational and experimental biology approach, we found that the nuclear factor FOXO3 is a crucial regulator of red blood cell production by coordinating the expression of many of the genes specific for terminal maturation of red blood cells. Furthermore we found that FOXO3 can even increase the production of normal red blood cells in culture raising the possibility that enhancing FOXO3 may have a therapeutic use. Our studies identify FOXO3 as a novel regulator of RBC enucleation and terminal erythropoiesis.

## Introduction

Erythropoiesis ensures the daily production of over 200 billion RBCs whose main function is to carry oxygen. Decreased production of RBCs is associated with many human disorders involving impaired erythroblast maturation. The generation of RBCs *in vitro* from embryonic stem cells or human-induced pluripotent stem cells (iPS cells) has been proposed to provide a cost-effective and safe blood supply. Despite recent development [1] achieving efficient production of functional RBCs has been hindered by incomplete knowledge of terminal erythroblast maturation.

Generation of RBCs involves the differentiation of hematopoietic stem cells into common megakaryocyte and erythroid progenitors, which give rise to lineage-restricted erythroid progenitors, erythroblasts, and ultimately erythrocytes. During the final stages of erythropoiesis, proliferation of erythroblasts is coupled with differentiation as terminally differentiating erythroblasts accumulate hemoglobin, reduce cell size, and condense their nuclei. Following enucleation, reticulocytes remodel their membrane and clear mitochondria and remaining organelles to transition into fully mature erythrocytes [2]. This complex process is controlled by integration of erythropoietin receptor (EpoR) signaling with the function of erythroid lineage-specific transcription factors including GATA–1, KLF–1 and TAL–1 (SCL) and their cofactors [3]. Despite recent progress [4–6], many questions remain unanswered regarding whether these factors function alone or together to control enucleation and/or to remove organelles, including mitochondria, during terminal erythroblast maturation. Increasing evidence suggest that FOXO3 cooperates with these factors and their requisite coregulators to control specific molecular/cellular steps that drive terminal erythroid maturation [4–6].

FOXO3 belongs to the FOXO family of Forkhead transcription factors composed in mammals of the highly related members FOXO1, FOXO3, FOXO4 and FOXO6. FOXOs are homeostatic maintaining factors implicated in many diseases including cancer, diabetes, and erythroid disorders [7–10]. FOXOs integrate fundamental biological processes through the regulation of cell cycle, oxidative stress, DNA damage responses, apoptosis, inflammatory responses, and metabolism [7,11]. FOXO genes have evolutionary conserved functions in stem cell maintenance and longevity [12–24]. Emerging evidence suggests that FOXO may also play a key role in tissue-tissue communication [7,25–27]. Among FOXO factors, FOXO3 is critical for normal and stress erythropoiesis [8–10,28–31]. This is evident as *Foxo3* mutant mice die rapidly when exposed to acute erythroid oxidative challenge [29]. Notably, FOXO3 expression and function increase progressively with erythroblast maturation [29,32,33]. Despite these findings, whether FOXO3 has any function in the regulation of terminal erythroblast maturation remains unknown.

Using an integrated systems and experimental biology approach, we demonstrate that FOXO3 is critical for the correct temporal expression of at least one third of the genes differentially expressed in normal maturing erythroblasts. Dysregulation of this subset of genes due to FOXO3 loss led to defects at distinct stages of terminal erythroblast maturation and RBC production. Our data demonstrate that FOXO3 is critical for erythroblast enucleation through polarization of the nucleus expulsion direction and is required for mitochondrial clearance. The transcriptomic analyses also revealed that increasingly maturing primary erythroblasts express immune-related transcripts whose expression is highly modulated upon loss of FOXO3. Collectively, these findings demonstrate that FOXO3 is an essential component of the transcriptional program that regulates terminal erythroblast maturation and required for the erythroblast enucleation process.

## Results

### Comparative transcriptomic analysis reveals potential new functions for FOXO3 in terminally maturing erythroblasts

**Fig 1:**
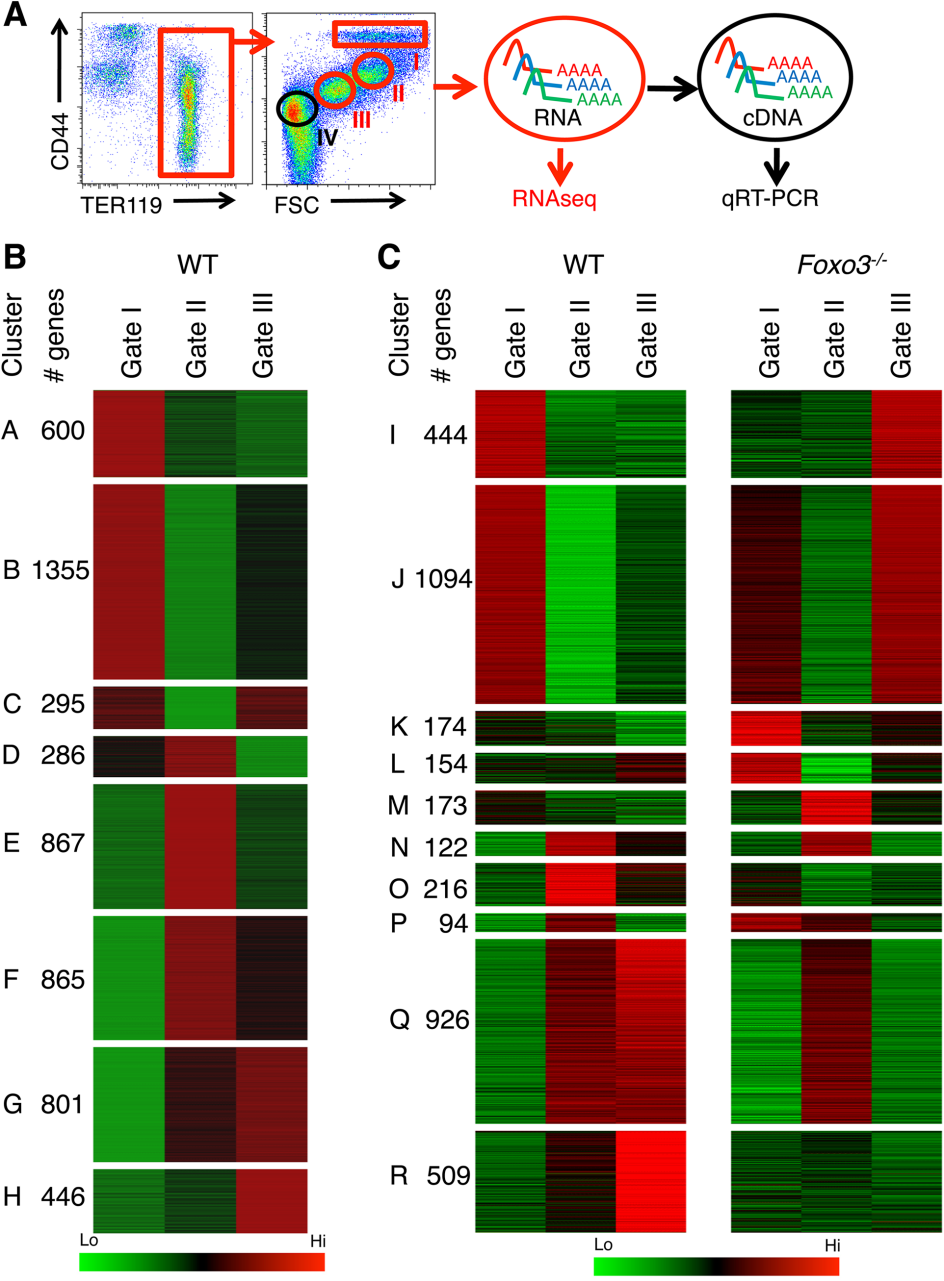
Deregulated gene expression in maturing *Foxo3* ^*-/-*^ erythroblasts. **(A)** Flow cytometry strategy used to FACS sort pro-, basophilic and polychromatic erythroblasts (Gates I to III respectively, in red) from wild type and *Foxo3* ^*-/-*^ bone marrow according to their TER119 and CD44 cell surface expression and forward scatter properties for RNA-Seq. Gate IV cells (depicted in black) are purified in subsequent experiments for experimental validation purposes. **(B)** Heatmap of differentially expressed genes in WT erythroblasts (low in green to high in red). Clustering of genes was performed according to their expression level in WT pro-, basophilic and polychromatic erythroblasts. Only the 5514 genes that varied at least 2 fold from pro- to polychromatic erythroblasts were used for clustering. **(C)** Heatmap of differentially expressed genes in WT versus *Foxo3* ^*-/-*^ erythroblasts at each gate (low in green to high in red). Clustering of the 3904 differentially expressed genes between WT and *Foxo3* ^*-/-*^ samples (amplitude ≥ 2) is shown. Amplitude was calculated as the difference between the same gates of WT and *Foxo3* ^*-/-*^ erythroblasts.

To investigate the FOXO3-regulated transcriptional program during erythroblast maturation we compared the transcriptome of adult bone marrow erythroid precursor populations of *Foxo3* ^*-/-*^ mice to that of wild type (WT) mice. Since immature erythroblasts accumulate in *Foxo3* mutant bone marrow [29], we reasoned that the relative accumulation of immature erythroblasts due to FOXO3 deletion might reflect a block in their terminal maturation. Immature erythroblasts were isolated using flow cytometry relying on the immunophenotype and forward scatter properties of erythroblasts according to Chen et al. [34]. The erythroid specific marker TER 119 was combined with CD44 and forward scatter, which both decrease during maturation, to resolve the progressive stages of erythroblast differentiation [34] (Fig 1A). This approach enabled the distinction and purification of three consecutive stages of maturation of erythroid populations (pro-, basophilic and polychromatophilic erythroblasts) as defined by gates I, II and III respectively (Fig 1A). Relatively pure subpopulations of erythroblasts as shown by morphological analysis (S1A Fig) were isolated using this gating strategy. RNA was isolated from the first three gates (Fig 1A, *depicted in red*) and deep sequencing analysis was conducted to compare wild type and *Foxo3* mutant erythroblast transcriptomes. In the morphological analysis and subsequent validation experiments, Gate IV cells (*depicted in black*) encompassing mainly reticulocytes that are enucleated cells preceeding mature red blood cells were also included (Fig 1A).

Erythroid-specific genes of wild type erythroblasts were grouped according to low, intermediate, or high levels of expression that was validated by qRT-PCR, which faithfully reproduced the levels and patterns of gene expression in each group (S1B Fig and S1 Table). Expression of globin genes was used as a control to confirm the identity of erythroblasts. Although the expression was too high to be reliably quantified by cufflinks software used for RNA-Seq analysis, qRT-PCR analysis demonstrated globin gene upregulation during erythroid maturation (S1C Fig and S1 Table). Analysis of wild-type erythroblasts revealed 5514 genes (S2 Table) with at least two fold differential expression between gates. These genes were grouped into eight clusters using k-means clustering (Fig 1B). Using Gene Ontology (GO) term analysis with FuncAssociate 2.0, signaling pathways and biological processes enriched in each of the clusters were delineated (S3 Table). The chromatin immunoprecipitation (ChIP) enrichment analysis tool, ChEA, [35,36] was used to identify potential transcription factors that may occupy the genes within each cluster (S4 Table). Cluster A, which grouped 600 genes that were continuously downregulated from Gates I to III, is enriched in inflammatory and apoptotic genes. In agreement with RNA-Seq analysis of human erythroblasts, genes continuously up-regulated during terminal erythroid maturation and clustered in G and H (801 and 446 genes respectively) were enriched for autophagy-related genes underscoring the importance of autophagy during erythroblast maturation [4,33,37]. Genes initially upregulated and then down-regulated were enriched for cell cycle-related processes, chromatin remodeling, DNA repair and mitotic genes (Clusters E and F, 867 and 865 genes respectively) (Fig 1B and S3 Table). Erythroid-specific genes including heme biosynthetic enzymes, iron metabolism, and erythroid membrane genes were distributed in clusters F, G, and H (S3 Table). Consistent with these results, known erythroid transcription factors including GATA–1, KLF–1, TAL–1, and the transcriptional co-activator EP300 (E1A binding protein p300) were associated with the sustained up-regulation in clusters G and H (S4 Table). Expression of Clusters E and F genes which were downregulated at Gate III, was associated with E2F4, E2F1, c-MYC transcription factors, and Cyclin D1 (CCND1), all known for regulating cell cycle progression. These data support previous findings [38] that erythroblasts continue to cycle at late stages of their maturation.

Immune-related genes were enriched in clusters B and C (1355 and 295 genes respectively) which include genes initially downregulated and then slightly upregulated in gate III (Fig 1B). As anticipated many inflammation-related genes, such as S100 Calcium Binding Protein A11 *(s100a11)*, interleukin 17 receptor α (*Il17ra)*, and immediate early response 2 (*Ier2*) were downregulated with maturation (S2A Fig). Furthermore, ChEA analysis implicated MYB and PU.1, known repressors of terminal erythroid maturation, as transcription factors regulating B and C gene clusters [39–41] (S4 Table). Accordingly, mRNA expression of MYB and PU.1 decreased during erythroid maturation (S2B Fig). Interestingly and consistent with previous findings [42,43], genes linked to several immune pathways, including several genes involved in the interferon response and lymphocyte activation pathways, were upregulated upon terminal erythroid maturation (clusters G and H) (Fig 1B). Expression of interferon-related genes including interferon regulatory factor (*Irf*7) and radical S-adenosyl methionine domain containing 2 *(Rsad2)* was validated by qRT-PCR which found these to be upregulated 2–15 fold upon maturation of bone marrow erythroblasts (S2C Fig). The corresponding proteins were also expressed in TER119^+^ erythroblasts that are negative for CD45 (TER119^+^CD45^-^) confirming the specificity of their expression and lack of non-erythroid cell contamination (S2D Fig). These observations suggest that these immune-related genes may have a specific function in terminal erythroid maturation.

### FOXO3 is a critical determinant of the erythroid cell transcriptome

For comparing WT and *Foxo3* mutant erythroblast expression profiles, genes were re-clustered. Genes with at least a two-fold differential expression between the same gate of WT and *Foxo3* mutant erythroblasts were reclustered for further analysis. Expression of 3906 genes (S5 Table), approximately 35% of the total expressed genes, was strikingly altered in *Foxo3* ^*-/-*^ erythroid precursors (Gates I to III). In agreement with the progressive increase in the expression and function of FOXO3 with erythroid maturation [29,31], the majority of differences in gene expression among gates I to III of wild type and *Foxo3* ^*-/-*^ erythroblasts were detected in Gate III (S3A and S3B Fig). Loss of FOXO3 led to both repression (clusters Q and R, 926 and 509 genes respectively) and induction (clusters I and J, 444 and 1094 genes respectively) of gene expression during erythroid maturation (Fig 1C). Immune-related pathways, including macrophage and neutrophil activation pathways enriched in clusters I and J, fell within programs that are normally repressed, but were aberrantly upregulated in the absence of FOXO3 (Figs 1C and S3C and S6 Table). These results indicate that loss of FOXO3 may enhance the expression of many inflammatory-related genes in maturing erythroblasts (S3C Fig), consistent with the anti-inflammatory function of FOXO3 [27,44]. The greatest impact of FOXO3 loss was exemplified by clusters Q and R, which consist of genes normally upregulated during erythroid maturation. Cluster R was enriched for autophagy and catabolic processes, while Cluster Q was enriched for heme biosynthesis, erythroid differentiation, nucleosome assembly, and DNA packaging-related processes (Fig 1C and S6 Table). Both clusters Q and R designate genes that are continuously up-regulated from Gates I to III. However, differences arise between the clusters from the loss of FOXO3. In the absence of FOXO3, the progressive upregulation of genes between Gates I to III is abrogated in cluster R. In contrast genes in cluster Q are not upregulated from Gates II to III. The stalled gene activation in Cluster Q suggests that FOXO3 is required for the transition to the gene expression profile characteristic of Gate III erythroblasts. The distinct pattern of clusters Q and R may reflect distinct modes of FOXO3 action for each respective gene cohort. Since 40% of the genes in both clusters are established targets of GATA–1, TAL–1, and/or KLF–1 transcription factors (S7 Table) [3], these results, consistent with previous findings [6], raise the possibility that FOXO3 cooperates with these factors to sustain gene transcription during terminal erythroid maturation.

### Defective autophagy in Foxo3^-/-^ erythroblasts impairs reticulocyte maturation

Autophagy was one of the main pathways highly up-regulated upon erythroblast maturation (Fig 1B, clusters G and H). However, autophagy-related genes were greatly dysregulated in *Foxo3* ^*-/-*^ mutant erythroblasts (Fig 1B, and S6 Table cluster R). Autophagy (or macro-autophagy) serves as a homeostatic mechanism that mediates the consumption of damaged or old cellular components, as well as the cellular remodeling that is associated with cell differentiation [45]. In erythroblasts, autophagy is implicated specifically in the clearance of mitochondria (mitophagy or selective mitochondrial autophagy) during terminal erythroid maturation [37,46,47].

**Fig 2:**
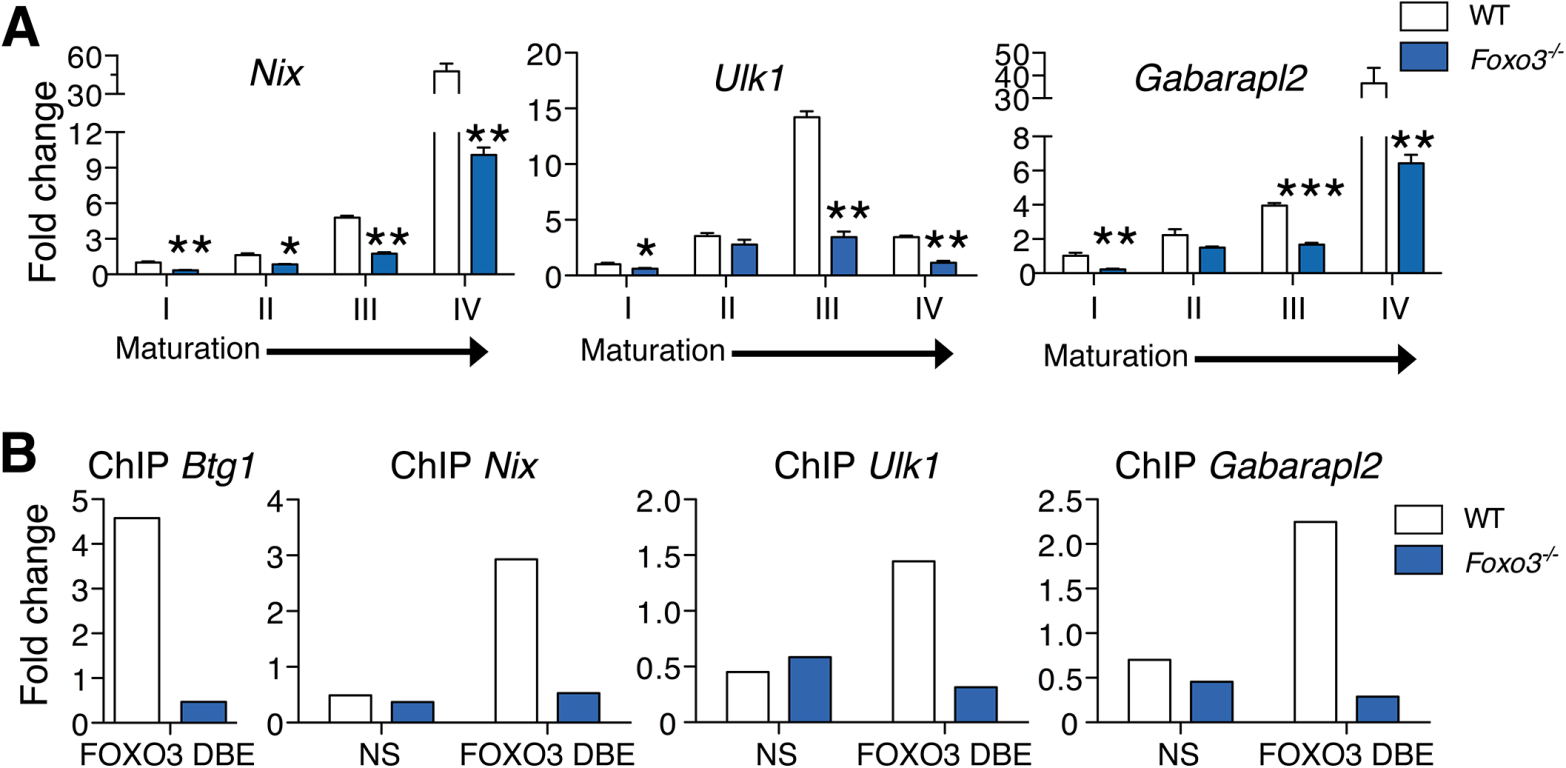
FOXO3 regulates expression of autophagy and mitochondrial removal related genes in erythroblasts. **(A)** Fluidigm microfluidic qRT-PCR expression analysis of autophagy genes in WT and *Foxo3* ^*-/-*^ Gates I to IV bone marrow erythroblasts. Quantification of target genes is relative to β actin. Results are mean ± SEM of 3 cDNAs, each generated from one mouse. *P < 0.05 **P < 0.01 ***P < 0.001, Student’s *t* test. **(B)** FOXO3 binding to Btg1, Nix, Ulk1 and Gabarapl2 regulatory regions as determined by ChIP in total TER119^+^ cells. Enrichment of putative FOXO3 DNA binding regions in Btg1 (positive control), Nix, Ulk1 and Gabarapl2 promoters was analyzed by qPCR and compared to regions with no known FOXO3 binding sites in wild type versus *Foxo3* ^*-/-*^ erythroid cells used as negative controls. Values were normalized to Ct values from total input. One representative of two experiments is shown. NS Not specific; DBE DNA binding element.

To evaluate the role of FOXO3 in regulating autophagy in primary erythroblasts, we validated expression of autophagy-related genes differentially expressed between wild type and *Foxo3* mutant erythroblasts (Figs 2A and S4A). The expression pattern of some of these genes, including Nix (Bnip3l) and Ulk1 (Fig 2A), was upregulated 15- to over 40-fold during late stages of bone marrow erythroblast maturation and reticulocyte formation. This finding is supported by the known function of these genes in mitochondrial removal [46–48]. The similar pattern of expression of other autophagy-related genes, including *Gabarapl2* (Gate–16), *p62*, *Atg14*, and *Pink1* (Figs 2A and S4A) suggests their involvement in erythroid maturation. In addition, autophagy genes, including *Gabarapl1* and *Map1lc3b* lie within the highly expressed gene cohort at all stages of erythroblast maturation indicating these genes may be involved in the homeostatic control of erythroid maturation (S1 Table). Consistent with this, the core autophagy genes *Atg5* and *Atg7* were expressed at similar levels throughout erythroid maturation relative to other autophagy genes examined (S4B Fig). However, expression of several autophagy-related gene transcripts was profoundly compromised in *Foxo3* mutant erythroblasts (Figs 2A and S4A), suggesting that these genes are potential direct targets of FOXO3 in erythroblasts. ChIP analysis revealed occupancy at *Btg1*, a known FOXO3 direct target [28] and several autophagy-related genes including *Nix*, *Gabarapl2* and *Ulk1* in wild-type, but not in *Foxo3*-deficient erythroblasts. Thus, FOXO3 occupied regulatory regions of these genes in primary erythroblasts *in vivo* (Fig 2B). Occupancy was not detected at upstream sequences lacking FOXO3 binding sites in these regulatory regions. Several of the genes shown in Figs 2A and S4A are direct FOXO3 transcriptional targets, including *Map1lc3b* and *Atg14* in non hematopoietic cells [49–51], while others including *Gabarapl2* were not known to be regulated by FOXO3 in any system. Interestingly, *Nix* and *Ulk1* are known regulators of erythroblast mitochondrial removal [47] [48].

**Fig 3:**
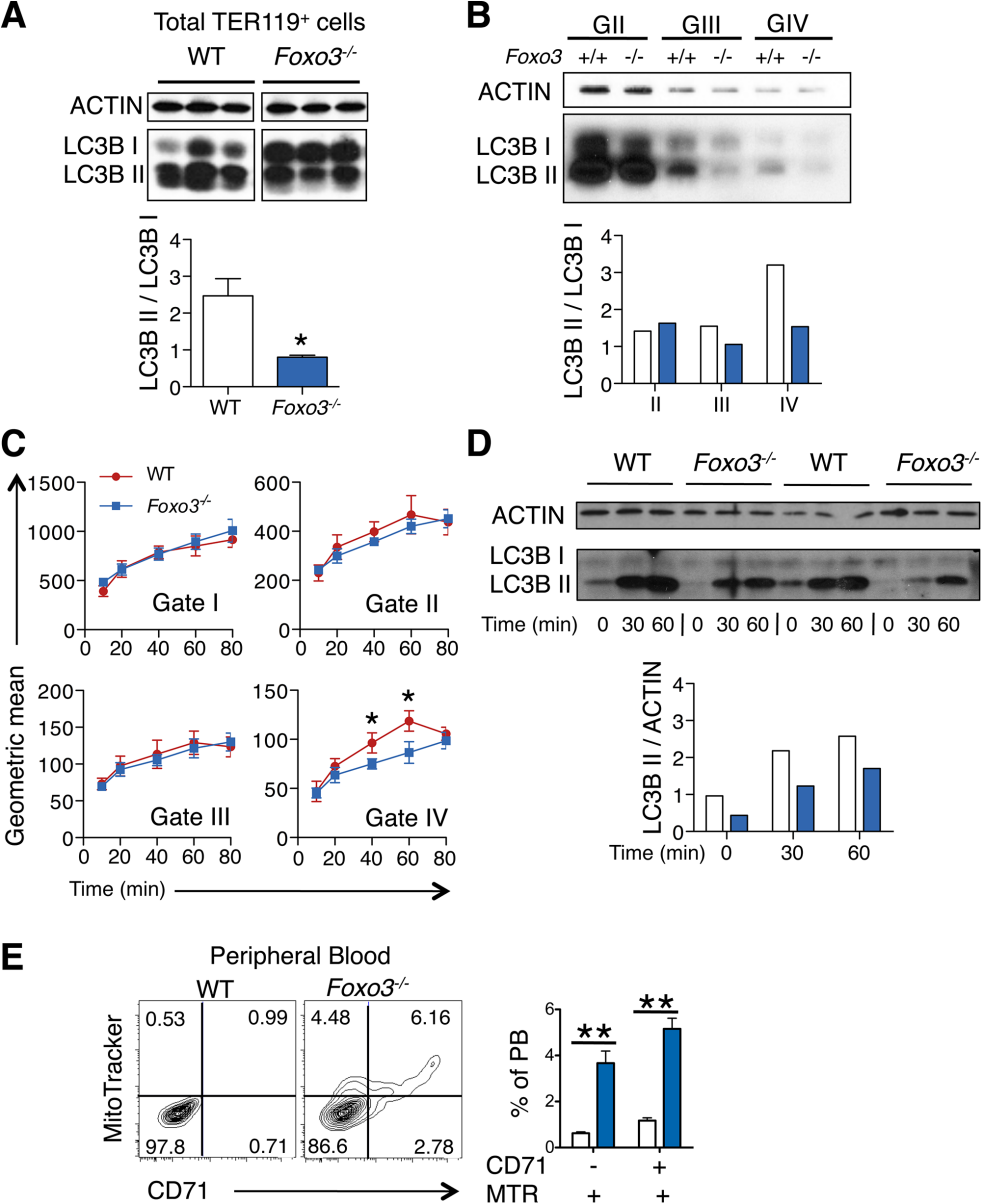
Autophagy and mitochondrial removal are impaired in *Foxo3* ^*-/-*^ erythroblasts **(A)** Western blot analysis of LC3B protein of WT and *Foxo3* ^*-/-*^ bone marrow TER119^+^ cells (n = 3 mice for each genotype). Quantification of the LC3BII/ LC3B-I ratio in one representative of two independent experiments is shown (bottom panel). **(B)** Western blot analysis of LC3B protein extracted from WT and *Foxo3* ^*-/-*^ bone marrow erythroblasts Gates II to IV (insufficient Gate I cell numbers for Western blot). Quantification of the LC3B-II/LC3B-I ratio is shown (panel below). **(C)** Autophagic flux in WT and *Foxo3* ^*-/-*^ bone marrow cells was analyzed by flow cytometry. Cells were cultured with chloroquine (50 μM) for the indicated time points and autophagosomes were detected by Cyto-ID in specific gates according to TER119, CD44 and FSC properties. Flux was calculated by subtracting the value obtained from the untreated sample to the value obtained at each of the different time points. Results are mean ± SEM of n = 3. One representative of three independent experiments is shown. **(D)** Aliquots of cell lysates from (**C**) at the indicated time points were subjected to Western blot analysis of LC3B showing two replicates. Quantification of the LC3B-II protein is normalized to total actin, and the relative accumulation of LC3B-II is quantified (bottom panel). **(E)** Flow cytometry analysis (left panels) and quantification (right panel, n = 4 in each genotype) of Mitotracker Red CMXRos in combination with CD71 surface expression of WT and *Foxo3* ^*-/-*^ peripheral blood. *P < 0.05 **P < 0.01 ***P < 0.001, Student’s *t* test.

We reasoned that the failure to upregulate autophagy gene expression might decrease autophagy during differentiation of *Foxo3*-mutant erythroblasts and compromise their terminal maturation. To test this, we measured the conversion of the soluble free form of Microtubule-associated protein 1 light chain 3B (LC3B-I) to the lipidated LC3B-II form, an essential step in autophagosome activation. Autophagosomes are generated as a result of sequential assembly and activation of autophagy-related proteins [45]. Once activated, they engulf the damaged organelle or proteins and fuse with lysosomes to degrade their cargo. The LC3B-II/LC3B-I ratio, an important indicator of autophagy [52], was decreased significantly in freshly isolated primary *Foxo3*-mutant erythroblasts under homeostatic conditions (Fig 3A), suggesting that autophagy was compromised in these cells. Further gate-by-gate analysis suggested that defective autophagy was mainly observed within *Foxo3* ^*-/-*^ gate III and IV erythroblasts (Fig 3B). To further ensure that these results reflected impairment in autophagosome formation and not abnormalities related to events upstream or downstream [53], we evaluated the autophagic flux in erythroblasts using an autophagosome-specific fluorescent probe [54]. Addition of chloroquine to bone marrow cultures blocked degradation and induced accumulation of autophagosomes in wild type and *Foxo3* ^*-/-*^ erythroblasts, enabling their measurement over time by flow cytometry and western blotting for LC3B-II (Fig 3C and 3D). *Foxo3*-deficient erythroblasts exhibited significantly reduced autophagic flux as compared to their wild type counterparts specifically in Gate IV erythroblasts that are highly enriched in reticulocytes (Fig 3C). The LC3B-II accumulation was also delayed in chloroquine-treated *Foxo3* ^*-/-*^ relative to wild type erythroblasts (Fig 3D). The reduced autophagy flux was mainly detected in late stage *Foxo3*-deficient erythroblasts, consistent with the alteration of autophagy-related gene expression in these cells (S4A Fig). Collectively, these results are consistent with the notion that FOXO3 controls autophagy at late, but not early, stages of erythroid maturation (Fig 3B and 3C).

We considered whether low levels of autophagy that are characteristic of *Foxo3* ^*-/-*^ erythroblasts suffice to support mitochondria removal during reticulocyte maturation. The frequency of circulating reticulocytes in *Foxo3*-mutant mice is increased to compensate for increased RBC destruction [29]. However, in *Foxo3*-mutants a significant fraction of mature RBCs are devoid of the transferrin receptor CD71 but remain positive for the mitochondria-specific MitoTracker Red probe (Fig 3E). This was further confirmed using a distinct mitochondria-specific probe MitoTracker Green (S4C Fig). These results indicate that despite their increased numbers in *Foxo3* mutant mice [29] reticulocytes retain mitochondria, delaying the complete maturation into RBCs. This increase is consistent with the failure to induce multiple autophagy-related genes in *Foxo3* ^*-/-*^ erythroblasts, which have been implicated specifically in mitophagy (Figs 2A and S4A). Nonetheless, the impact of impaired autophagy on mitochondrial removal in *Foxo3*-mutant reticulocytes appeared to be relatively modest, suggesting that compensatory mechanisms might be involved. Alternatively these results might reflect the contribution of distinct autophagy pathways controlling mitochondrial removal in erythroid cells [55–57].

### FOXO3 is essential for erythroblast enucleation

**Fig 4:**
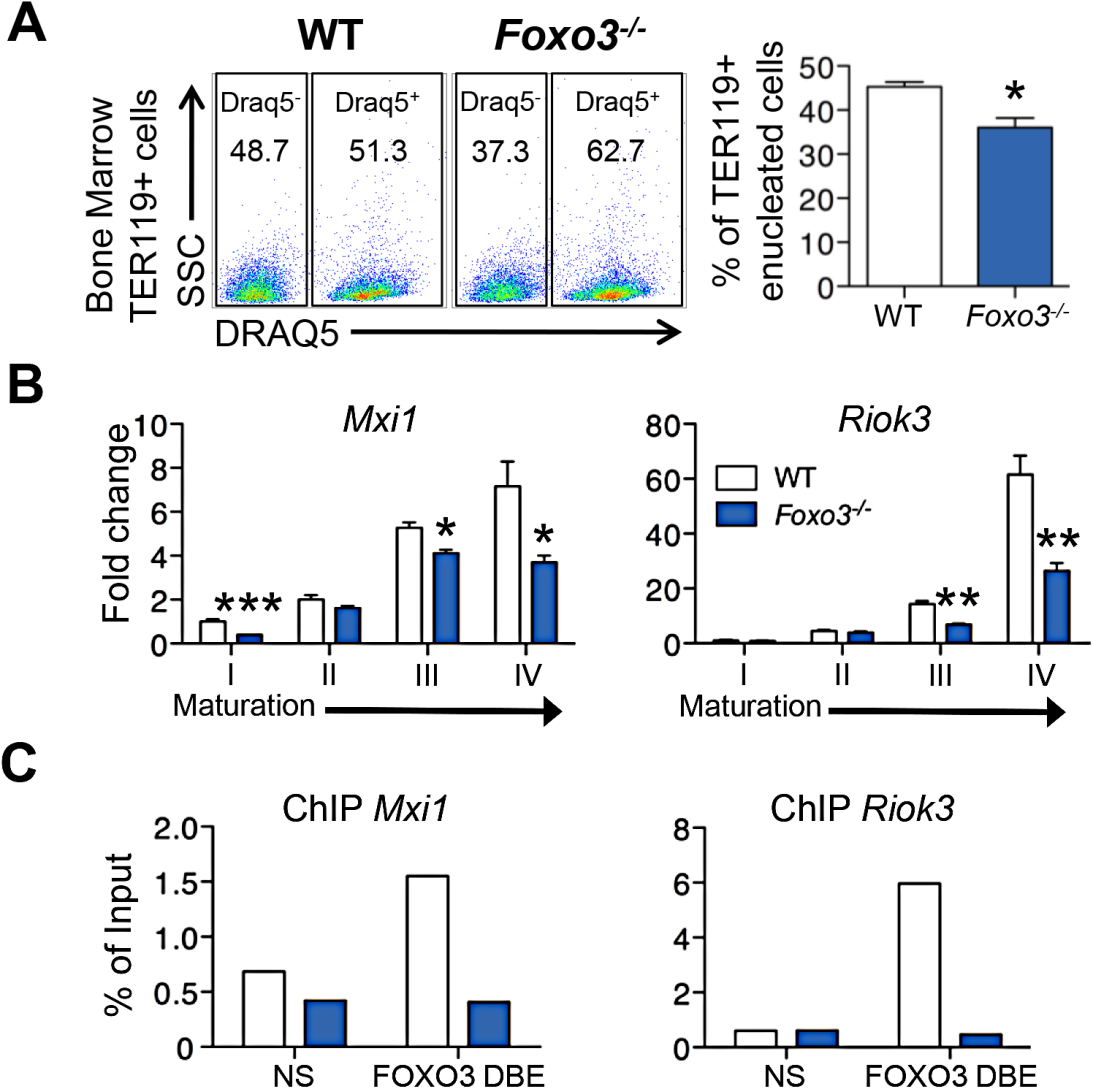
Impaired enucleation-related gene transcription in *Foxo3* ^*-/-*^ bone marrow erythroblasts. **(A)** WT and *Foxo3* ^*-/-*^ bone marrow TER119^+^ erythroblasts were analyzed for DRAQ5 staining by flow cytometry. The percentage of enucleated TER119^+^ cells is shown (lower panel). Results are mean ± SEM of n = 3; one representative of four different experiments is shown. **(B)** QRT-PCR expression analysis by Fluidigm microfluidics technology of enucleation-related genes in WT and *Foxo3* ^*-/-*^ Gates I to IV bone marrow erythroblasts. Quantification of target genes is normalized to β actin. Results are mean ± SEM of 3 cDNAs. **(C)** FOXO3 occupation of Mxi1 (left) and Riok3 (right) regulatory regions as determined by ChIP. Enrichment of putative FOXO3 DNA binding regions in Mxi1 and Riok3 promoters was analyzed by qPCR and compared to regions with no known FOXO3 binding sites. Values were normalized to Ct values from total input. One representative of two different experiments is shown. NS Not specific; DBE DNA binding element. *P < 0.05 **P < 0.01 ***P < 0.001, Student’s *t* test.

Key steps in RBC formation include chromatin condensation and expulsion of the nucleus (enucleation) from late-stage erythroblasts [2] [58] [59]. We discovered that many genes involved in nucleosome assembly and DNA packaging-related processes were downregulated in *Foxo3*-mutant erythroblasts (Fig 1C, cluster Q, S6 Table), raising the possibility that FOXO3 controls chromatin condensation and/or enucleation. Using DRAQ5, a fluorescent probe that binds DNA *in vivo*, we found that there were significantly fewer enucleated *Foxo3*-mutant bone marrow erythroblasts than wild type cells (Figs 4A and S5A). QRT-PCR expression analysis of cluster Q showed that genes implicated in chromatin condensation and/or enucleation *Mxi1*, *Riok3*, *Smarca4*, *Trim58*, *Rac GTPase I* and *II* [60–63] were all reduced at distinct stages of *Foxo3*-mutant erythroblast maturation (Figs 4B and S5B). Among these, *Riok3* and *Trim58* transcripts were upregulated 60- and over 120-fold, respectively, during differentiation of normal bone marrow erythroblasts, suggesting these genes may function beyond enucleation during terminal erythroblast maturation (S5B Fig). In contrast, expression of *RacGTPase I* and *II* decreased with maturation of normal bone marrow erythroblasts. ChIP of endogenous FOXO3 in wild type, but not in *Foxo3* mutant, bone marrow erythroblasts revealed occupancy at regulatory regions of *Mxi1* and *Riok3* (Fig 4C). Thus, these genes similar to the autophagy genes examined earlier may also be directly regulated by FOXO3 in erythroblasts.

**Fig 5:**
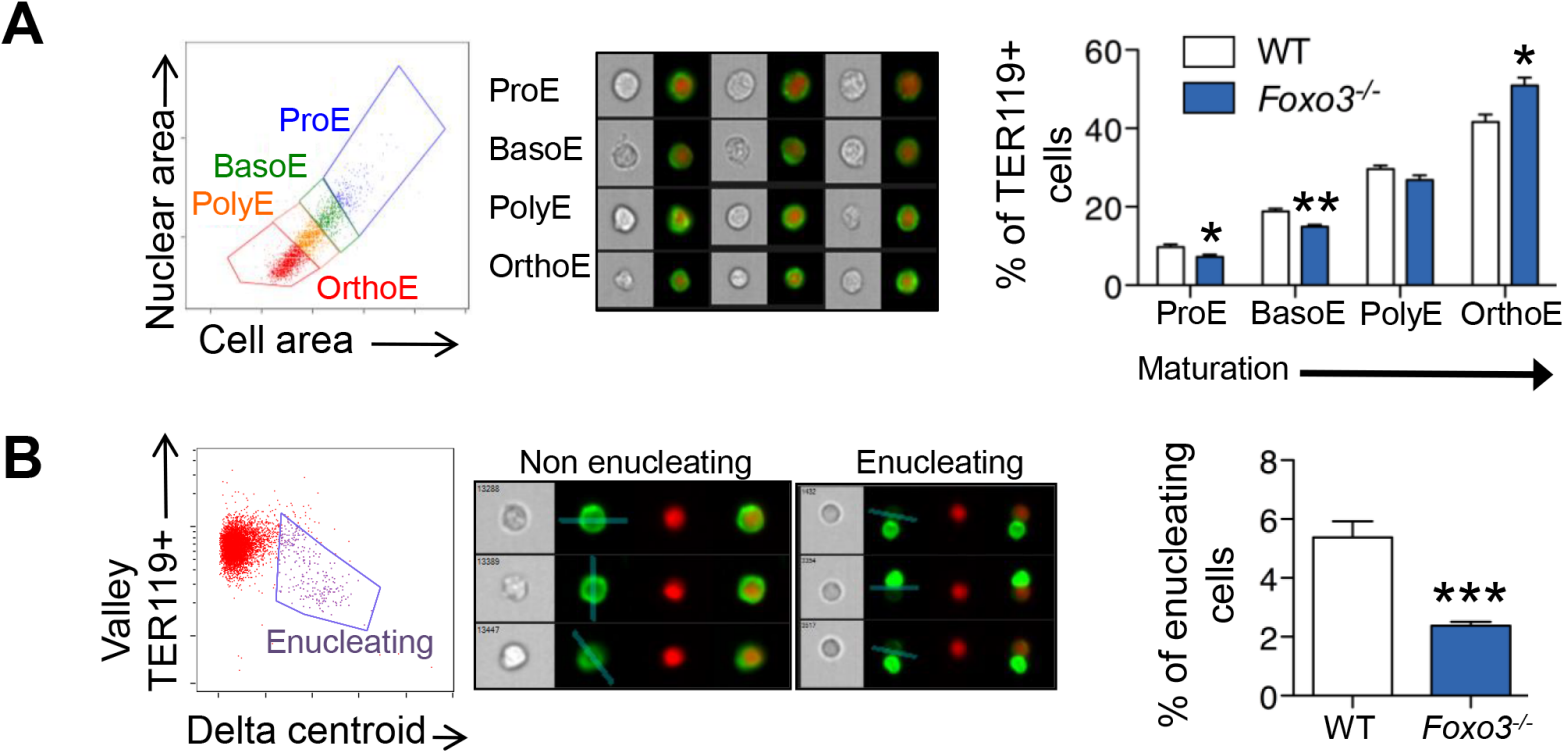
Defective *Foxo3* ^*-/-*^ terminal erythroblast maturation. **(A)** Delineation of erythroblasts by imaging flow analysis based on decreasing cell and nuclear size (left). Images of typical cells (middle panel) in bright-field (gray) and composite image of TER119 (green) and DRAQ5 DNA stain (red). Results are mean ± SEM of n = 4. (**B**) Gating of enucleating cells from the orthochromatic erythroblasts (in **A**) based on difference between the center of the nuclear stain and TER119 stain (Delta centroid on X) and a decreasing TER119 signal in the valley mask (highlighted in blue on TER119 image) which goes through the lowest signal point and thus marks the boundary between the reticulocyte and pyrenocyte. Cells with no signal in this mask are eliminated as close doublets. Results are mean ± SEM of n = 4. *P < 0.05 **P < 0.01 ***P < 0.001, Student’s *t* test.

We used imaging flow cytometry to develop mechanistic insights into the potential enucleation defect in *Foxo3* mutant erythroblasts. As described previously, [58,64] TER119^+^ erythroblasts can be segregated according to their cell and nuclear size into progressive stages of maturation (pro-, basophilic, polychromatic and orthochromatic erythroblasts) (Fig 5A). While this analysis confirmed the increase in erythroid precursors described previously in *Foxo3* mutant bone marrow (S5C Fig) [29], the analysis also revealed that the accumulation occurs at the orthochromatic stage of *Foxo3* mutant erythroblast maturation (Figs 5A and S5C). Prior to enucleation, late-stage erythroblasts displace the nucleus from its central location. The percentage of orthochromatic erythroblasts containing an asymmetrically positioned nucleus was quantified using the delta centroid, a measure of the distance between the center of the cell body and center of the DRAQ5-stained nucleus [58]. This analysis revealed a lower percentage of enucleating erythroblasts in the *Foxo3*-mutant versus wild-type bone marrow (Fig 5B).

**Fig 6:**
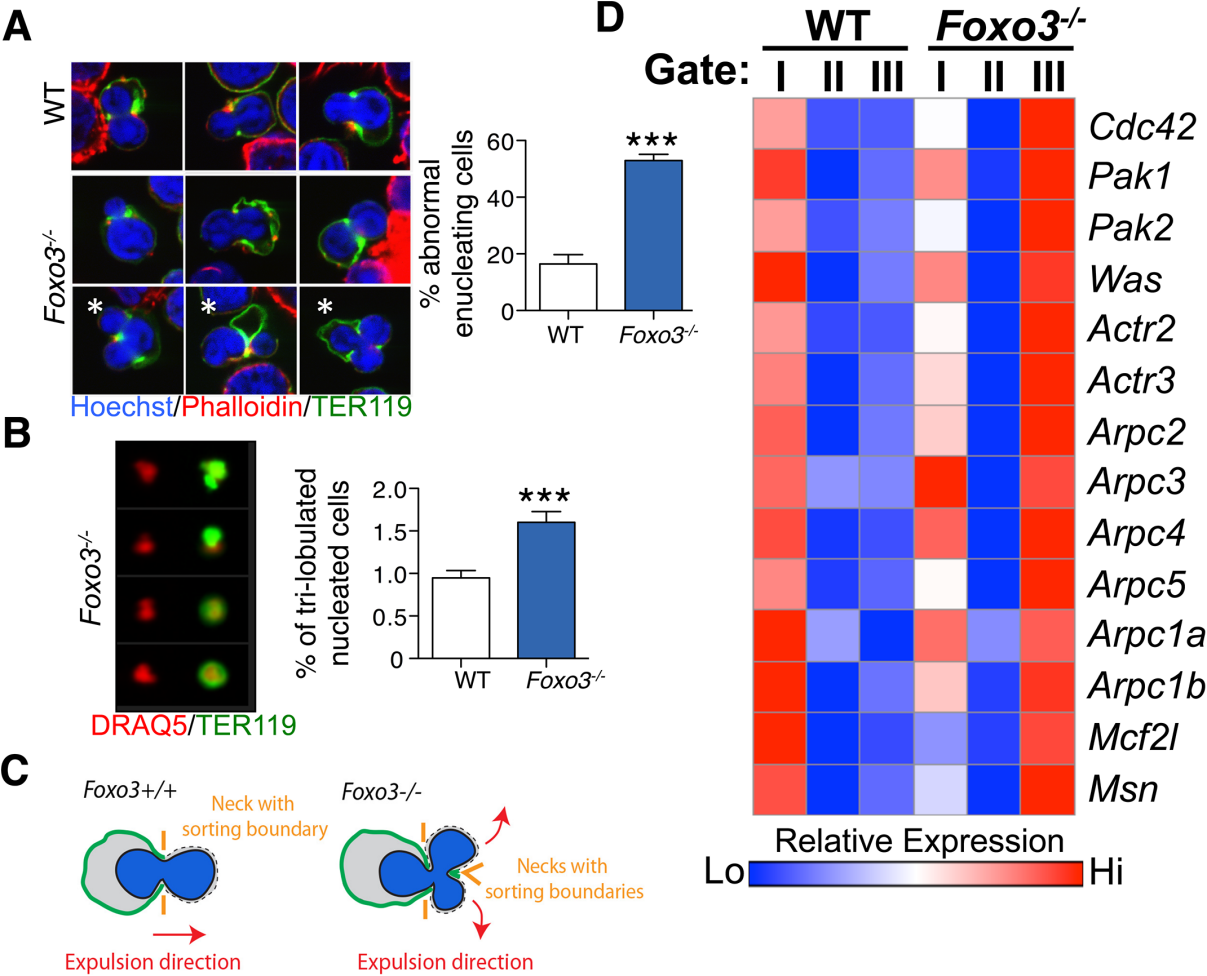
Defective enucleation in *Foxo3* ^*-/-*^ bone marrow erythroblasts. **(A)** Enucleation was analyzed by immunofluorescence of freshly isolated bone marrow cells from WT (n = 5) and *Foxo3* ^*-/-*^ (n = 3) mice using anti-TER119 antibody (green), Rhodamine Phalloidin (red) and Hoechst (blue). Images were obtained by confocal microscopy and abnormal enucleating cells counted. Representative images of enucleating cells are shown, with white asterisks denoting abnormally enucleating cells. At least 10 enucleating cells were counted per bone marrow and the results indicate the percentage of abnormal enucleating cells in each bone marrow as mean ± SEM. **(B)** Quantification of abnormal nuclei within the orthochromatic faction of WT and *Foxo3* ^*-/-*^ erythroblasts by imaging flow cytometry. Abnormal nuclei were defined as having high 3-fold symmetry of the nucleus [64]. Representative images of normal and abnormal nuclei from *Foxo3* mutants are shown are shown. Results are mean ± SEM of n = 4. *P < 0.05 **P < 0.01 ***P < 0.001, Student’s *t* test. **(C)** Model for the impact of loss of FOXO3 on the enucleation process. **(D)** Heatmap of RNA-Seq data of CDC42-related gene cluster (both upstream and downstream of CDC42) implicated in polarity and actin polymerization in Gates I-II and III of *Foxo3* wild type and mutant erythroblasts.

To investigate how the FOXO3 loss impacts enucleation, we conducted a high-resolution morphological analysis of bone marrow erythroblasts. As shown in Fig 6A, confocal analysis revealed wild-type enucleating erythroblasts by the presence of a gap in the bright TER119 membrane staining as described previously [65]. Wild type enucleating erythroblasts displayed a dumbbell-shaped nucleus, with a neck located at the TER119 sorting boundary of the nascent reticulocyte. This ensures that cells use a single direction for nuclear extrusion. In contrast, *Foxo3* mutant erythroblasts exhibited multiple nuclear necks accompanied by multiple sorting boundaries with each lobe extruding in a different direction away from the nascent reticulocyte. This unique pattern suggested defective polarization of *Foxo3* mutant erythroblasts during enucleation (Fig 6A). Accordingly, 48% of the *Foxo3* ^*-/-*^ enucleating erythroblasts exhibited abnormal enucleation morphologies. These results were verified by imaging flow cytometric analysis by distinguishing orthochromatic erythroblasts with tri-lobular nuclei from normal extruding nuclei (Fig 6A–6C). This analysis demonstrated that erythroblasts with tri-lobular nuclei were remarkably more frequent in *Foxo3* ^*-/-*^ versus wild type bone marrow (Fig 6B amd 6C). These results indicate that FOXO3 is critical for enucleation and may control erythroblast polarization/nuclear positioning as a prelude to enucleation. Consistent with this notion, several genes implicated in cytoskeleton organization, cell polarization and cytokenesis, are deregulated in *Foxo3* ^*-/-*^ erythroblasts (Figs 6D, S5C and S6A). Notably, the small RhoGTPase CDC42-related gene cluster that regulates cytoskeleton organization and cell polarization [66,67] is aberrantly upregulated in *Foxo3* ^*-/-*^ erythroblasts (Fig 6D).

**Fig 7:**
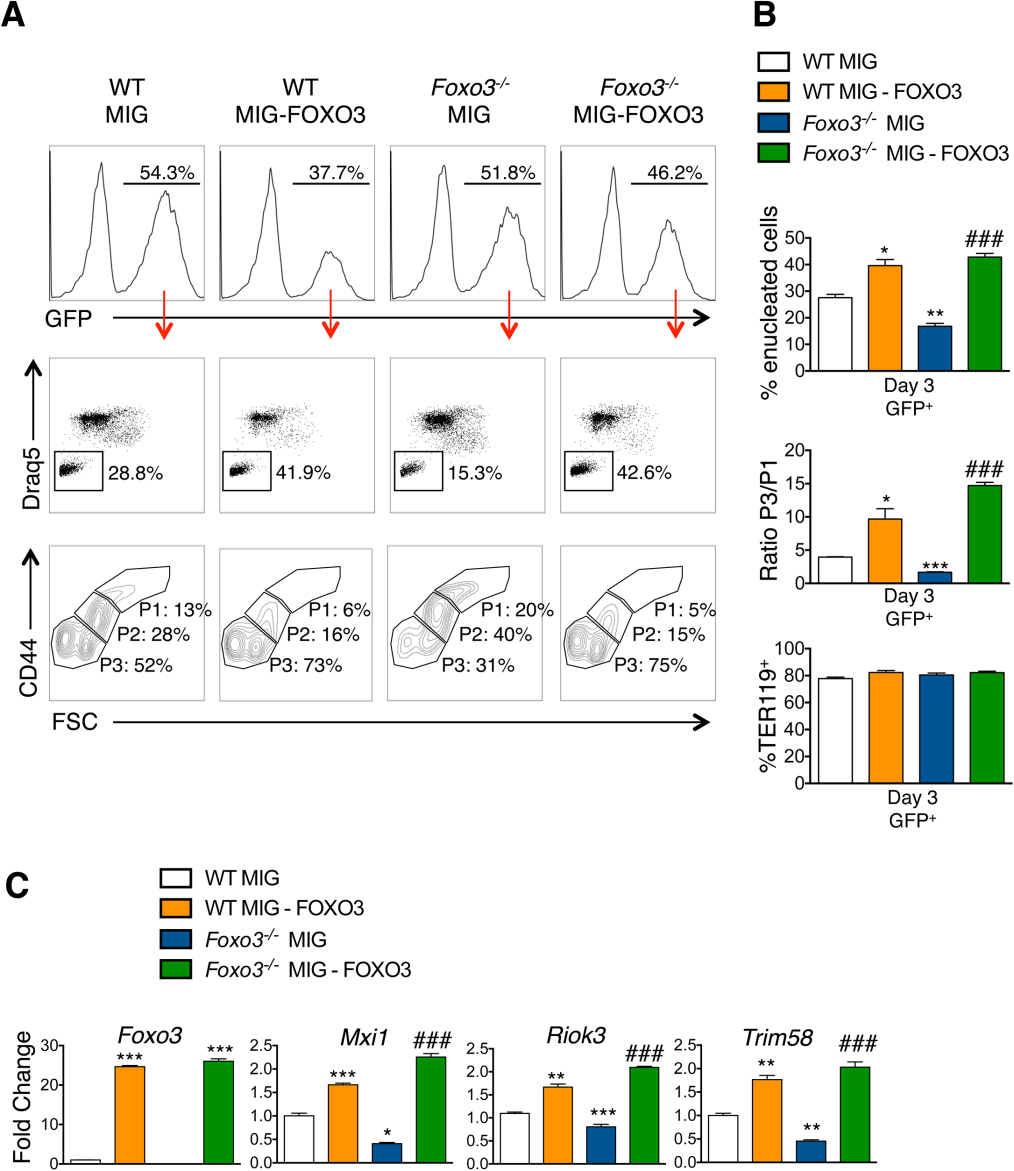
Ectopic expression of FOXO3 rescues terminal maturation and enucleation in *Foxo3* ^*-/-*^ erythroblasts. **(A)** WT and *Foxo3* ^*-/-*^ bone marrow erythroid progenitors were expanded and transduced with either the MIG vector overexpressing FOXO3 (MIG-FOXO3) or the MIG vector alone, transferred to erythroid differentiation medium and incubated for additional three days of maturation after which cells were analyzed by flow cytometry. Upper panels show the percentage of transduced cells within each culture condition after maturation. Middle panels show representative FACS plots for each condition indicating the percentage of enucleated cells within transduced (GFP^+^) cells. Bottom panels show representative FACS plots of maturing erythroblasts. P1 represents the most immature and P3 the most mature erythroid populations. **(B)** Quantification of enucleation in GFP^+^ cells shown as mean of percent enucleated ± SEM of n = 3 (Upper panel). Quantification of overall maturation kinetics is based on CD44 and FSC parameters as a ratio between the most mature population (P3) over the least mature population (P1) (middle panel). TER119^+^ cells (%) is shown (bottom panel). **(C)** qRT-PCR expression analysis of enucleation-related genes in WT and *Foxo3* ^*-/-*^ GFP^+^ FACS sorted cells after 3 days of maturation. Quantification of genes is normalized to β actin and relative to WT cells transduced with MIG. Results are mean ± SEM of duplicates of 3 cDNAs. *P < 0.05 **P < 0.01 ***P < 0.001 compared to WT MIG, ^*###*^ *P <* 0.001 compared to *Foxo3* ^*-/-*^ MIG; Students *t* test.

To investigate whether FOXO3 might directly regulate enucleation, we examined the capacity of FOXO3 to rescue enucleation in *Foxo3* mutant erythroblasts. MSCV-IRES-GFP (MIG) or MIG-FOXO3 were ectopically expressed in wild type and *Foxo3* ^*-/-*^ bone marrow-derived erythroid progenitors and subjected to *in vitro* maturation by adapting a previously established protocol, for bone marrow erythroblasts [68] (Fig 7A). Gene expression patterns of *Gata1*, *Pu*.*1*, *and Hbb-b2*, were examined over 3 days of maturation and validated that they resemble *in vivo* erythroblast maturation patterns (S7A Fig). Flow cytometry analysis enabled resolution of the most immature erythroblast populations (*ex vivo* P1), exhibiting high CD44 levels and large size, compared to the most mature erythroblast population (*ex vivo* P3), exhibiting low CD44 levels and small size. The ratio of P3 to P1, termed maturation index here, reveals the extent of maturation at day 3 of culture. *Foxo3* ^*-/-*^ erythroblasts show an overall decrease in their ability to mature (Fig 7A and 7B) [29]. Importantly, ectopic expression of FOXO3 in mutant erythroblasts rescued their defective maturation without modulating the total number of TER119 cells (Fig 7A–7C). In addition, the last stage of maturation was specifically impaired in *Foxo3* mutant erythroblasts (P3/P2) at ∼ 50% (± 0.046, n = 3) of that of wild type (P3/P2) cells and rescued by ectopic expression of FOXO3 (Fig 7A, bottom panels). Furthermore, a similar degree of rescue by ectopic FOXO3 expression was observed when comparing the very last stages, S2 (enucleated) and S1 (nucleated) mature erythroblasts in culture, where the majority of enucleation occurs (S7B Fig). In fact, overexpression of FOXO3 rescued the enucleation capacity of *Foxo3* ^*-/-*^ erythroblasts to levels similar or higher than that of wild type controls in *ex vivo* maturation cultures (Fig 7A and 7B). In support of a potential direct FOXO3 control of enucleation the ectopic expression of FOXO3 increased the expression of *Mxi1*, *Riok3*, and *Trim58* (Figs 7C and S7C). Notably, overexpression of FOXO3 further improved enucleation, the maturation index, and expression of enucleation related genes in WT erythroblasts (Figs 7A–7C and S7B). Ectopic expression of FOXO3 also improved expression of autophagy-related genes in *Foxo3* ^*-/-*^ erythroblasts (S7C Fig).

## Discussion

Our studies show that FOXO3 has critical physiological functions at key steps of terminal erythroid maturation. While FOXO3 is required for enucleation and for subsequent mitochondrial clearance in reticulocytes, FOXO3 loss impacts more profoundly upon the enucleation process. Enucleation is a critical limiting step in erythroid maturation [2]. The removal of the nucleus results from the culmination of a multi-step process that starts early in differentiation and encompasses chromatin condensation, cell polarization, formation of contractile actin ring and ultimate enucleation [2]. Our studies demonstrate that FOXO3 is essential for normal enucleation to occur. FOXO3 directly controls the expression of several critical regulators of enucleation, including *Riok3* and *Xpo7*, important regulators of chromatin condensation [49,60,69], as well as *Mxi1*, implicated in contractile actin ring formation [60]. FOXO3 also potentially controls *Trim58* expression that is a gene critical for erythroblast nuclear polarization and/or extrusion [63] (Figs 4 and S5B). Loss of FOXO3 severely compromised erythroblast polarization and resulted in multilobed nucleated erythroblasts in agreement with FOXO3 regulation of genes implicated in cell polarity and cytoskeleton organization (Figs 4–6, S5 and S6). The aberrant gene expression of the CDC42 network suggests that FOXO3 may mediate polarization or the cytoskeleton organization of enucleating erythroblasts, which are potentially important for the chromatin condensation and/or nuclear expulsion (Figs 4–6). These abnormalities are likely to be direct consequence of FOXO3 loss and not due to processes resulting from developmental lack of FOXO3, as reintroduction of FOXO3 normalized the defective enucleation within a short period of time in culture (Fig 7). Future studies will address the precise mechanism of FOXO3 function in this process. PI3-kinase, an upstream negative regulator of FOXO3 in erythroblasts [70], is required for erythroblast maturation [71,72] and polarization during enucleation *in vitro* [73]. However, signals other than PI3-kinase/AKT are likely to override the PI3-kinase repression of FOXO3 activity during erythroblast enucleation *in vivo* [18]. It is therefore probable that both PI3-kinase and FOXO3 control erythroblast terminal maturation and enucleation.

FOXO3 is an established regulator of autophagy [25,74–76]. Here we demonstrated that FOXO3 is critical for removal of mitochondria during terminal erythroid maturation. We found that FOXO3 regulates transcription of autophagy-related genes in maturing erythroblasts and FOXO3 loss compromised autophagosome formation and autophagy, reduced autophagic flux in maturing erythroblasts and impaired mitochondrial clearance in RBCs (Figs 2 and 3). Despite the profound reduction in autophagy gene expression, specifically at late stages of erythroblast maturation, and the negative impact on autophagosome formation, the influence of FOXO3 loss on mitochondria clearance was relatively modest (Fig 3E). Together these findings indicate that under homeostatic conditions, the relatively low autophagy gene expression is sufficient to maintain clearance of mitochondria in FOXO3 mutant erythroblasts. These results support the notion that multiple autophagy pathways might regulate mitochondrial clearance in erythroid cells [55–57].

In erythroid cells, FOXO3 amplifies the capacity of GATA–1 to induce autophagy gene expression [4,5]. Consistent with this, loss of FOXO3 led to notable decrease in the expression of many of autophagy-related genes in primary maturing erythroblasts (Fig 2) despite endogenous GATA–1 expression (S7A Fig). These results indicate that GATA–1 induction of autophagy gene expression in terminally maturing primary erythroblasts may be compromised in the absence of FOXO3.

**Fig 8:**
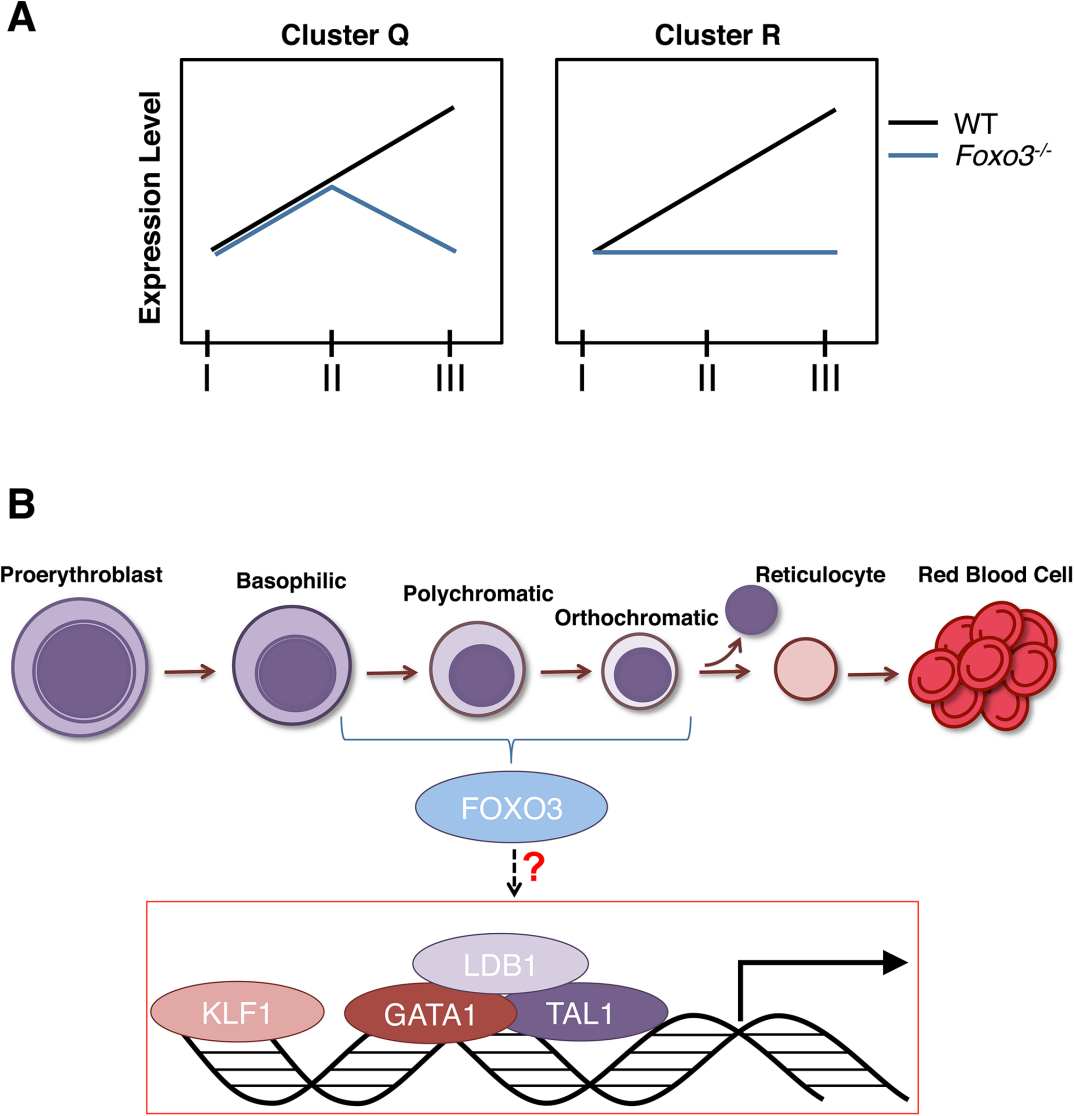
Model. **(A)** Depiction of expression of clusters Q and R genes in *Foxo3* ^*-/-*^ versus wild type erythroblasts. Cluster Q is enriched for nucleosome assembly, heme biosynthesis, and DNA packaging-related processes while cluster R is enriched for autophagy and catabolic processes. **(B)** Model for gene expression in terminally maturing erythroblasts. Complexes of core erythroid transcription factors regulate the genetic programs required for maturation of the initial erythroblast stages. These transcription factor complexes may also induce *Foxo3* expression in immature erythroblasts. In turn, FOXO3 cooperates with these factors to sustain and/or enhance the erythroid transcriptional program during the later stages of terminal maturation.

Our unbiased genome-wide analysis revealed that, in addition to the approximately 600 genes that have been categorized as erythroid specific [6], more than 1000 genes, including genes involved in cell cycle, autophagy and immune-modulation are upregulated during terminal erythroid maturation. Loss of FOXO3 impaired significantly the expression of over 65% of these genes. Furthermore, FOXO3 loss limited to various degrees (clusters Q and R) the increase in expression of several GATA1, TAL1 and/or KLF–1 targets during terminal erythroid maturation. These results (Fig 1C) combined with the ChEA prediction (S7 Table), support a model in which FOXO3 is required to activate and/or enhance (cluster R), or sustain (cluster Q) gene expression during terminal erythroid maturation (Fig 8). As further support for this potential role of direct FOXO3 transcriptional activation of Cluster Q and R genes, we analyzed a list of direct FOXO3 targets determined by ChIP-seq [77] in a colon carcinoma cell-line. This comparison shows a significant number of overlap with cluster Q and R genes, suggesting that these genes are direct FOXO3 targets (S8A Fig, S8 Table). A sizeable number of genes do not overlap; this is most likely due to a difference in cell type and species. Consistent with previous findings [4–6,77–80] these results support a model (Fig 8) in which erythroid transcription factor complexes induce *Foxo3* gene expression in immature erythroblasts which in turn cooperates with these factors to sustain and/or enhance the erythroid transcriptional program during terminal maturation. A similar model has been proposed for the induction of expression of interferon regulatory factor IRF2 and IRF6, which subsequently cooperate with GATA–1 and TAL1 transcription factors to establish the adult human erythroid program [81]. Collectively, these findings raise the possibility that this mode of regulation of gene expression might be a common mechanism during erythroid differentiation and maturation. Cooperation of these transcription factors with FOXO3 is likely to be critical for terminal erythroblast maturation. Nonetheless, in the absence of ChIP-Seq data, we are unable to determine with certainty all genes that are direct products of FOXO3 cooperation with other transcription factors in maturing erythroblasts.

Taken together our findings demonstrate that FOXO3 activity critically regulates progressive stages of terminal primary erythroblast maturation. As FOXO3 activity is critical for the correct temporal gene expression in maturing erythroblasts, we predict that abnormal FOXO3 expression/function may significantly influence erythroid disorders as has been reported specifically for hemoglobinopathies [8–10]. Since post-translational modifications of FOXO3 are the main determinants of FOXO3 output, analysis of FOXO3 function in addition to its expression will be critical in the context of disease. Future studies should elucidate whether modulations of FOXO3 activity influence the efficiency of RBC production *in vitro* or in disorders of erythroid cells *in vivo*.

## Materials and Methods

### Mice

The generation and genotyping of mice (on C57Bl6 genetic background) were performed as previously described [14,31]. WT and *Foxo3* ^*-/-*^ mouse littermates (8 to 12 weeks old) of *Foxo3* heterozygous intercrosses were used in all experiments. Protocols were approved by the Institutional Animal Care and Use Committee of Mount Sinai School of Medicine.

### Library preparation and mRNA sequencing

Total bone marrow cells from WT and *Foxo3* ^*-/-*^ mice (n = 3) were collected in IMDM supplemented with 15% FBS and each genotype combined. Cells from the two samples were pre-incubated with 10% rat serum, stained for TER119 and CD44 cell surface markers and three erythroid populations comprising proerythroblasts (Gate I), basophilic erythroblasts (Gate II) and polychromatic erythroblasts (Gate III) were FACS sorted as previously described [34]. Total RNA was isolated using the RNeasy Micro Kit (Qiagen) and mRNAseq libraries were prepared using the Illumina True Seq RNA prep kit following manufacturer’s instructions. Samples were sequenced in parallel lanes in a Hi Seq™ 2000 platform (Illumina) to obtain more than 10^7^ single end 100bp reads per sample.

### RNA-seq read processing

The RNA-seq reads were mapped to mouse genome build mm9 using Tophat v2.0.3 [82] with default parameters. The mapped reads were processed to calculate the FPKM (fragments per kilo-base per million reads) and identify differentially expressed genes using Cufflinks v1.3.0 [83]. The statistics for data processing is shown in (S9 Table).

### Pathway analysis of differentially expressed genes at distinct maturation stages

For WT analysis, only genes that fulfilled both of the following criteria were selected: (1) to show a FPKM > 1 in at least one of the three populations (pro-, baso- or polychromatic erythroblasts) and (2) to have an amplitude equal or above two. Amplitude was calculated as the maximum difference in expression between all three gates (e.g. Gate I vs Gate II, Gate II vs Gate III or Gate I vs Gate III). The resulted 5514 genes were then normalized using Z-score and clustered (Cluster 3.0 software k-means). For the comparison between WT and *Foxo3* ^*-/-*^ samples we followed the same strategy as stated above. Only those genes that displayed a FPKM ≥ 1 in at least one of the six samples and an amplitude ≥ 2 were considered as differentially expressed between WT and *Foxo3* ^*-/-*^ samples. In this case, amplitude was calculated by computing gene expression differences between WT and *Foxo3* ^*-/-*^ at each particular gate (e.g. WT Gate I vs. *Foxo3* ^*-/-*^ Gate I) and only genes with a two-fold difference in at least one of the comparisons were selected. The selected 3904 genes were then normalized using Z-score and clustered as indicated above.

### RNA Isolation and qRT–PCR

FACS-sorted cells were washed once in PBS and directly diluted in RLT buffer after centrifugation. Total RNA was isolated using the RNeasy Micro and Mini kit (Qiagen) same method used for RNA-seq. First-strand cDNA was synthesized using the SuperScript IITM Reverse Transcriptase (Invitrogen). Quantitative RT-PCR was performed using SYBR Premix Ex TaqTm II (Tli RNase H Plus) (Takara, #RR820A) with technical duplicates using an ABI Prism 7900 HT Cycler (Applied Biosystems). Gene-specific primers spanning intron-exon boundaries were designed using Primer-Blast (NCBI). Primers used in Fluidigm microfluidics technology experiments were designed by Fluidigm. The PCR cycle parameters were as follows: 95°C for 10’ followed by 40 cycles at 95°C for 15” and 60°C for 1’. Results were obtained with the Sequence Detection System 4.2 software (Applied Biosystems) and further analyzed by the 2-ΔΔCt method. β actin was used as a loading control. Results are shown as fold-change relative to wild type controls. Primer specific sequences are listed in S10 Table.

### Flow cytometry

Bone marrow single cell suspensions were obtained and washed in IMDM + 15% FBS. Cells were pre-incubated with 10% rat serum and stained with anti-CD44-APC (#559250 BD) and anti-TER119-PE (#553673 BD) antibodies. For DRAQ5 (#DR50200 Biostatus) analysis cells were stained for CD44-PE (#553134 BD) antibody and TER119-Biotin (#553672 BD) followed by Streptavidin-PE-Cy7 (#557598 BD). After washing, cells were incubated with DRAQ5 DNA binding dye (1/1000 dilution in PBS + 2% FBS) at 37°C and washed. Samples were analyzed in a FacsCanto flow cytometer (BD). Data was analyzed by FlowJo software (Treestar).

### Analysis of mitochondrial clearance

For MitoTracker Green or Red (#M7514, #M7512 Molecular Probes) analyses cells were pre-incubated with 10% rat serum, stained with anti-CD71-APC (#17–0711 eBiosciences) and incubated with mitotracker (20nM for 20 min at 37°C). Cells were then washed and analyzed by FACS.

### Autophagy flux

Autophagy flux was measured by flow cytometry using the Cyto-ID autophagy detection kit (Enzo Life Sciences). Briefly, WT and *Foxo3* ^*-/-*^ bone marrow cells were preincubated with 10% rat serum and stained with TER119-PE and CD44-APC antibodies on ice. After washing, cells were resuspended at 10^6^ cells/ml with 0.5 ml of IMDM supplemented with 15% FBS, 2 U/ml EPO and 50 ng/ml SCF in 24 well plates. Cells were cultured for 2 h at 37C° and chloroquine (50 μM) was added for 0, 10, 20, 40, 60, or 80 min. Cells were then kept on ice, washed twice in cold PBS + 5% FBS and resuspended in Cyto-ID solution consisting of 0.5 ml of PBS + 5% FBS + 0.25 μl of Cyto-ID. Cells were incubated for 30 min at room temperature in the dark, washed once with cold PBS + 5% FBS. Autophagosome content was determined by measuring the Cyto-ID (FITC) geometrical mean for each erythroid subpopulation. Autophagy flux was calculated by subtracting time 0 value from each of the other time points. All time points were analyzed for each bone marrow sample.

### Imaging flow cytometry

Bone marrow cells were pre-incubated with 10% rat serum and stained with TER119-FITC, CD71-PE, GR-1-EF450, and DRAQ5 (all from ebioscience). After washing, cells were fixed with 4% paraformaldehyde for 20 min at room temperature. Cells were centrifuged (30 seconds at 2000g), washed with PBS and centrifuged again. Images were acquired with an ImageStreamX and analyzed using IDEAS 6.0 software (Amnis/EMDmilipore). Single, focused, TER119^+^, Gr1^-^ cells were further analyzed for cells size (area TER119 morphology mask), nuclear size (area DRAQ5 morphology mask). Enucleating cells were enumerated from TER119^+^ small cell and nuclear size cells (orthochromatic cells- See Fig 3D) by increased asymmetry of nucleus (delta centroid X/Y of TER119 morphology mask and DRAQ5 morphology mask) and a boundary of low signal between the emerging pyrenocyte and reticulocyte parts of the enucleating cell (TER119 intensity in Valley mask). Lobular shaped nuclei were identified using the 3-fold symmetry feature on the DRAQ5 morphology mask.

### Fluidigm—96.96 Dynamic Array IFC

For Fluidigm dynamic array performance, specific target amplification (STA) was performed according to the manufacturer´s protocol (PN 100–3488 B1). Briefly, cDNA was pre-amplified using the TaqMan PreAmp Master Mix (Applied Biosystems) for the 96 genes of interest. The amplification parameters were as follows: 95°C for 10’, followed by 12 cycles at 95°C for 15” and 60°C for 4’. After STA, Exonuclease I treatment was performed as recommended by the manufacturer. Briefly, Exonuclease I and Exonuclease I buffer (New England Biolabs) were added to the STA samples, and samples were then incubated for 30’ at 37°C, followed by the enzyme inactivation at 80°C for 15’. Finally, to load the dynamic array IFC, samples were prepared with the SsoFast EvaGreen Supermix with Low ROX (Bio-Rad) and 20x DNA Binding Dye Sample Loading Reagent (Fluidigm). Primers were diluted with Assay Loading Reagent (Fluidigm) and DNA Suspension Buffer (Teknova). After priming the 96x96 chip in the IFC Controller MX, samples and primers were loaded into their respective inlets. The chip was then loaded by the IFC Controller MX. The chip was run following the GE 96x96 PCR+Melt v2.pcl protocol in the Biomark using the Data Collection Software (Fluidigm). Results were obtained with the Fluidigm Real-Time PCR Analysis software (Fluidigm) and further analyzed by the 2-ΔΔCt method. β actin was used as a loading control. Results shown as fold-change relative to Gate I wild type controls. Primer specific sequences are listed in S10 Table.

### Confocal fluorescence microscopy

Total bone marrow cells were fixed in 4% paraformaldehyde (PFA) overnight on ice, washed with PBS, permeabilized with 0.3% Triton–100 for 20 min, and blocked in 3–4% BSA, 1% goat serum in PBS overnight at 4°C. Cells were then stained in solution as described previously [65] with anti-TER119-Alexa488, Rhodamine- Phalloidin and Hoechst. After washing in PBS, cells were cytospun into slides, coverslipped and images acquired on a Zeiss LSM780 confocal fluorescence microscope with a 100X/1.4 N.A. objective using zoom 1 or 2. The entire cytospin area was systematically scanned to avoid bias in collection. Images were processed using Volocity 6.1.1 and Adobe Photoshop, and images constructed in Adobe Illustrator.

### Western blot analysis

Equal WT and *Foxo3* ^*-/-*^ cell numbers were resuspended directly in Laemmli sample buffer with DTT. Samples were then boiled at 95°C for 10 min and kept at -80°C until used. Electrophoresis was performed on a 15% SDS-PAGE and transferred to a PVDF Immobilon-P membrane (Millipore). Membranes were blocked with 5% BSA in PBS 0.1% Tween–20 and incubated overnight at 4°C in 1% BSA in PBS + 0.1% Tween–20 with either anti-LC3B (#3868 Cell Signaling) or anti-actin (sc–1616 Santa Cruz) antibodies and then incubated with the appropriate secondary antibodies conjugated to HRP at 1/5000 for 1 h at room temperature. Membranes were washed and developed using the ECL reagents (Pierce) with Blue sensitive films (Crystalgen). Films were then scanned and measurements were made using the Multi-Gauge software from Fujifilm following the manufacturer’s instructions. For flux measurements, TER119^+^ cells were isolated and plated in IMDM supplemented with 15% FBS, 2 U/ml EPO and 50 ng/ml SCF and kept in culture for 2 h. Chloroquine (50 μM) was then added to the cultures for the indicated time. Cells were then collected, washed in PBS and resuspended directly in Laemmli sample buffer and analyzed by Western blot.

### Chromatin Immunoprecipitation (ChIP)

ChIP was carried out in WT total bone marrow TER119^+^ cells as previously described [17]. Briefly, cells were cross-linked in 0.4% formaldehyde in PBS, and lysed (10 mM Tris-HCl pH8.0, 10 mM NaCl, 0.2% NP40). Lysate was sonicated for 30 cycles of 30 s on/30 s off at 4C° using a Bioruptor Standard sonication device (Diagenode). The cell lysate was then diluted in ChIP dilution buffer (20 mM Tris-HCl, pH 8.0, 2 mM EDTA 150 mM NaCl, 1% Triton, 0.01% SDS) and incubated at 4C° overnight with anti-FOXO3a antibody (Millipore #07–702) and Magna ChIPTM Protein A+G magnetic beads (Millipore #16–663). Beads were then washed (20 mM Tris-HCl, pH 8.0, 2 mM EDTA, 50 mM NaCl, 1% Triton, 0.1% SDS) and recovered. The antibody-protein-DNA complexes were reverse cross-linked for DNA isolation and quantitative PCR (qPCR) analysis. *Foxo3* ^*-/-*^ TER119^+^ cells were used as negative controls. Putative binding sites were located using MatInspector from Genomatix (http://www.genomatix.de/). Primer specific sequences are listed in S11 Table. See S12 Table for all antibodies.

### Erythroblast expansion and differentiation culture

Bone marrow cultures were performed using a modified protocol of [68]. Briefly, WT and *Foxo3* ^*-/-*^ bone marrow cells were enriched for hematopoietic progenitors using the EasySep Mouse Hematopoietic Progenitor Cell Enrichment Kit (#19756 Stemcell Technologies) and expanded at < 2x10^6^ cells/ml in non-treated 24-well plates with erythroid expansion medium. Erythroid expansion media consists of Stem Span SFEM (StemCell Technologies) supplemented with 2 U/ml human recombinant EPO (Amgen), 100 ng/ml SCF (PreproTech), 40 ng/ml insulin-like growth factor–1 (PreproTech), 10^−6^ M dexamethasone (D2915; Sigma), 0.4% cholesterol mix (Gibco) and 1% penicillin/streptomycin (Gibco). Two days later, cells were washed twice with PBS and plated at a concentration <1x10^6^ cells/mL in erythroid differentiation medium consisting of IMDM supplemented with 2 U/ml EPO, 100 ng/ml SCF, 10% Serum replacement (Invitrogen), 5% Platelet-Derived Serum, glutamine, MTG (1.27 μl in 10 ml of 1:10 dilution) and 10% Protein-Free Hybridoma Media. Cells were incubated in maturation medium for 3 days, with additional media added at day 3.

### Retroviral production and transduction

Retroviral supernatants of MIG and MIG-FOXO3 were produced as previously described [29,72]. Bone marrow expanding erythroid progenitors were resuspended in retroviral supernatants (multiplicity of infection of 10) on retronectin-coated dishes in expansion medium for 12 h. Suspension cells were then removed and only attached cells were collected by incubation with PBS and 5 mM EDTA at 37C° for 15 min. Cells were then washed, resuspended in expansion medium and incubated for another 24 h. Cells were then washed twice in PBS and cultured with erythroid differentiation medium as detailed above.

## Supporting Information

S1 Fig RNA-Seq analysis of bone marrow erythroblast transcriptome at progressive stages of maturation **(A)** Wright Giemsa staining (right panels) of cytospins of FACS (left panels) sorted bone marrow TER119^+^ WT and *Foxo3* ^*-/-*^ erythroblasts from Gates I to IV. **(B)** RNA-seq reads were processed with Cufflinks to calculate the FPKM (fragments per kilo-base per million reads; upper panels). Selected genes from the low, medium and high range of expression validated by qRT-PCR are shown (lower panels). QRT-PCR results show ΔCt corrected by β actin. **(C)** QRT-PCR expression analysis by Fluidigm microfluidics technology of the indicated globin genes in bone marrow WT and *Foxo3* ^*-/-*^ Gates I to IV erythroblasts. Quantification of target genes is relative to β actin. Results are mean ± SEM of 3 cDNAs, each generated from one mouse. *P <0.05; Student’s *t* test.
(PDF)

S2 Fig Modulations of immune-related pathways during erythroid maturation. **(A)** qRT-PCR analysis of immune-related genes found to be downregulated over terminal erythroid maturation. Quantification of target genes is normalized to β actin and relative to expression within Gate I. **(B)** QRT-PCR gene expression analysis in WT bone marrow erythroblasts. **(C)** Validation of expression of immune-related genes found to be upregulated with erythroblast maturation in bone marrow CD45^-^ Ter119^+^ fractions segregated by CD44 expression and FSC. Results are mean ± SEM of 3 cDNAs, each generated from one mouse. **(D)** Western blot expression analysis of IRF7 and RSAD2 in CD45^-^TER119^+^ FACS sorted bone marrow cells (n = 2 mice) as compared to total bone marrow (BM) cells (from right lane mouse).
(PDF)

S3 Fig Loss of FOXO3 leads to abnormal expression of immune related genes during erythroid maturation. **(A)** The number of differentially expressed genes between WT and *Foxo3* ^*-/-*^ erythroblasts at each gate during terminal erythroid maturation is shown together with the expression of *Foxo3* in that particular Gate. **(B)** Venn diagram showing the overlap between the genes differentially expressed at each gate between WT and *Foxo3* ^*-/-*^ erythroblasts. In total, 3904 distinct genes are differentially expressed. **(C)** QRT-PCR expression analysis of several immune-related genes differentially expressed between WT and *Foxo3* ^*-/-*^ bone marrow Gates I to IV erythroblasts grouped in cluster J in Fig 1C. Expression data for *Ier2*, *Il17rα*, and *S100a11* are from the same experiment in S2A Fig, with the addition of data from *Foxo3* ^*-/-*^ erythroblasts. Quantification of target genes is relative to β actin. Results are mean ± SEM of 3 cDNAs, each generated from one mouse. *P < 0.05; Student’s *t* test.
(PDF)

S4 Fig Autophagy gene expression and activity are impaired in maturing *Foxo3* ^*-/-*^ erythroblasts. **(A-B)** QRT-PCR expression analysis of autophagy genes **(A)** including core autophagy genes **(B)** in WT and *Foxo3* ^*-/-*^ Gate I to Gate IV erythroblasts. Quantification of target genes is normalized to β actin and relative to WT Gate I erythroblasts. Results are mean ± SEM of 3 cDNAs, each generated from one mouse. *P < 0.05; Student’s *t* test. **(C)** Flow cytometry analysis (left panels) and quantification (right panel, n = 4 in each genotype) of Mitotracker® Green in combination with CD71 surface expression of WT and *Foxo3* ^*-/-*^ peripheral blood. *P < 0.05 **P < 0.01 ***P < 0.001, Student’s *t* test.
(PDF)

S5 Fig Defective *Foxo3* ^*-/-*^ erythroid enucleation. **(A)** Quantification of total number of WT and *Foxo3* ^*-/-*^ bone marrow TER119^+^ DRAQ5^-^ cells. Results are mean ± SEM of BM cells from three mice per genotype. **(B)** QRT-PCR expression analysis of genes implicated in chromatin condensation and enucleation in WT and *Foxo3* ^*-/-*^ bone marrow Gates I to IV erythroblasts. Quantification of target genes is normalized to β actin. Results are mean ± SEM of 3 cDNAs, each generated from one mouse. **(C)** Quantification of total numbers of bone marrow WT and *Foxo3* ^*-/-*^ pro, basophilic, polychromatic, and orthochromatic erythroblasts (from two femurs and tibias). Results are mean ± SEM of 4 mice per genotype. *P < 0.05, **P < 0.01, ***P < 0.001; Student’s *t* test.
(PDF)

S6 Fig Altered expression of genes implicated in cytokinesis and polarity in *Foxo3* ^*-/-*^ erythroblasts. **(A)** QRT-PCR expression analysis of genes implicated in cytokinesis from FACS sorted WT and *Foxo3* ^*-/-*^ erythroblasts from Gates I to IV. Quantification of target genes are normalized to β actin and relative to either WT Gate I. Results represent mean ± SEM of 3 cDNAs, each generated from one mouse. *P < 0.05, **P < 0.01; Student’s *t* test. ND; not done.
(PDF)

S7 Fig Ectopic expression of FOXO3 rescues the expression of autophagy-related genes in *Foxo3* ^*-/-*^ erythroblasts. **(A)** QRT-PCR validation of erythroid gene expression after three days of maturation. WT and *Foxo3* ^*-/-*^ BM cells were extracted and subjected to erythroid maturation. At least 10^5^ cells were collected at each day and used to generate cDNA. Quantification of target genes is normalized to β actin and relative to WT erythroblasts at Day 0. Results represent mean ± SEM, n = 3. *P < 0.05, **P < 0.01; Student’s t test. **(B)** Representative FACS plots of GFP^+^, TER119^+^ maturing erythroblasts from Fig 7, with gates S1 and S2, which segregate the P3 population into more (S2) and less (S1) mature populations (left panels). Ratio of the S2 to S1 frequencies (right Panel). Results represent mean ± SEM, n = 3. *P < 0.05 compared to MIG-transduced WT cells, ^*##*^ *P <* 0.05 compared to MIG-transduced *Foxo3* ^*-/-*^ cells; Student’s *t* test. **(C)** QRT-PCR expression analysis of autophagy genes from cultured erythroblasts transduced with empty vector (MIG) or MIG-FOXO3 at day 3 of maturation is shown. GFP positive cells were FACS sorted. Quantification of target genes is normalized to β actin and relative to WT erythroblasts at day 0. Results represent mean ± SEM, n = 3. *P < 0.05 compared to MIG-transduced WT cells, ^*#*^ *P <* 0.05 compared to MIG-transduced *Foxo3* ^*-/-*^ cells; Student’s *t* test.
(PDF)

S8 Fig Overlap between FOXO3 ChIP-seq data and cluster Q and R genes. A list of genes considered to be directly activated by FOXO3 based on ChIP-seq peaks and RNA polymerase II occupancy from [77] (Red) of human DLD1 colon adenocarcinoma cell line was compared with genes from cluster Q and R (Blue). The data is displayed as a Venn diagram with overlapping gene shown in purple. A Chi squared test was performed to determine significance, with expected values based on an estimated number of total genes in the mouse genome as 23000. Chi squared equals 68.571 and the two-tailed P value is less than 0.0001.
(PDF)

S1 Table Raw RNAseq Data. (XLSX)

S2 Table Clustering of differentially expressed genes during WT erythroblast maturation. (XLSX)

S3 Table GO term Enrichment of WT clusters. (XLSX)

S4 Table ChEA analysis of WT clusters. (XLSX)

S5 Table Clustering of genes differentially expressed between WT and *Foxo3* ^*-/-*^ erythroblast maturation. (XLSX)

S6 Table GO term Enrichment of WT vs. *Foxo3* ^*-/-*^ clusters. (XLSX)

S7 Table ChEA analysis of WT vs. *Foxo3* ^*-/-*^ clusters. (XLSX)

S8 Table Overlap of FOXO3 direct targets with clusters Q and R (XLSX)

S9 Table Statistics of RNAseq reads. (XLSX)

S10 Table qRT-PCR and Fluidigm primers. (XLSX)

S11 Table ChIP primers. (XLSX)

S12 Table Antibodies. (XLSX)
