# Supplementary figures and images for: A Systems Approach Identifies Essential FOXO3 Functions at Key Steps of Terminal Erythropoiesis
Source: PLoS Genet. 2015 Oct 9;11(10):e1005526. doi: 10.1371/journal.pgen.1005526 (PMC4599908; doi:10.1371/journal.pgen.1005526)

# Figure S1

**A**

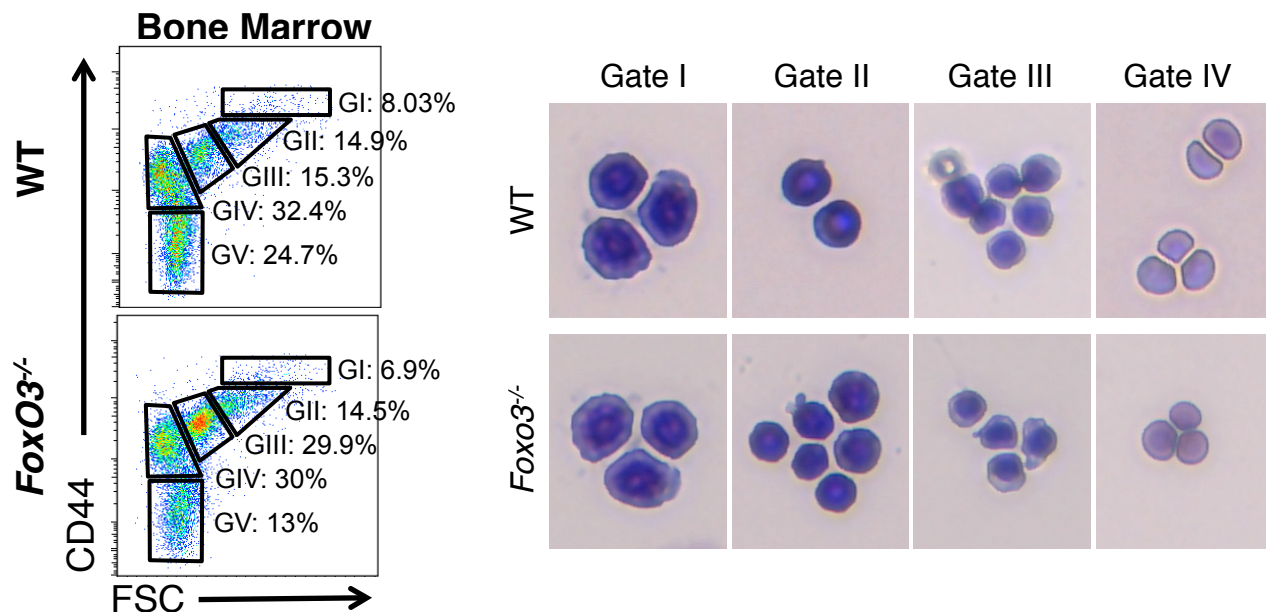

**B**

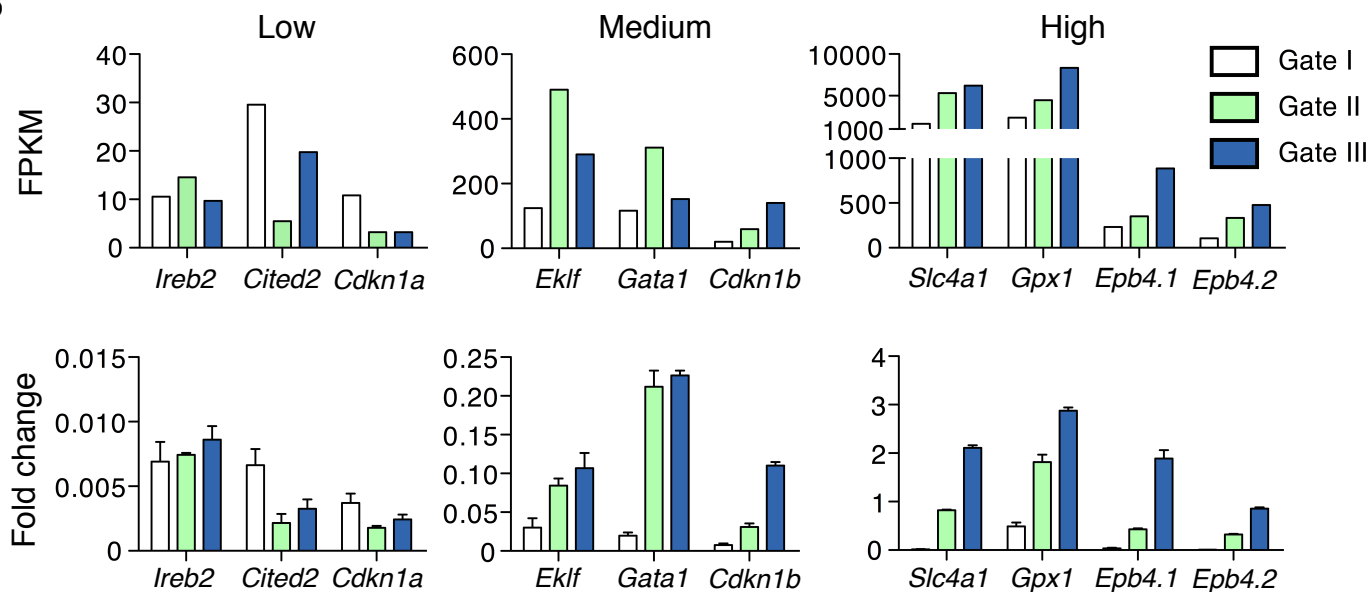

**C**

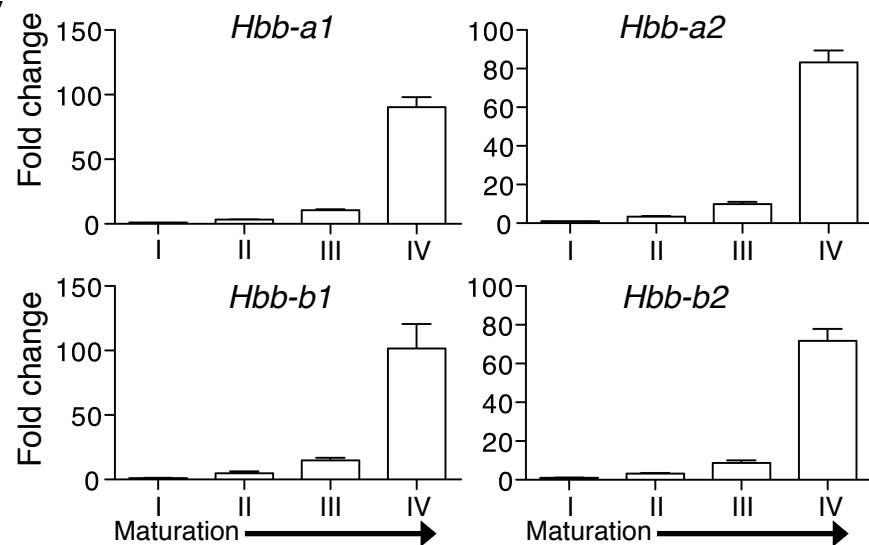

Supplement: S1 Fig — (A) Wright Giemsa staining (right panels) of cytospins of FACS (left panels) sorted bone marrow TER119+ WT and Foxo3 -/- erythroblasts from Gates I to IV. (B) RNA-seq reads were processed with Cufflinks to calculate the FPKM (fragments per kilo-base per million reads; upper panels). Selected genes from the low, medium and high range of expression validated by qRT-PCR are shown (lower panels). QRT-PCR results show ΔCt corrected by β actin. (C) QRT-PCR expression analysis by Fluidigm microfluidics technology of the indicated globin genes in bone marrow WT and Foxo3 -/- Gates I to IV erythroblasts. Quantification of target genes is relative to β actin. Results are mean ± SEM of 3 cDNAs, each generated from one mouse. *P <0.05; Student’s t test. (PDF) [file pgen.1005526.s001.pdf]

**Figure S2**

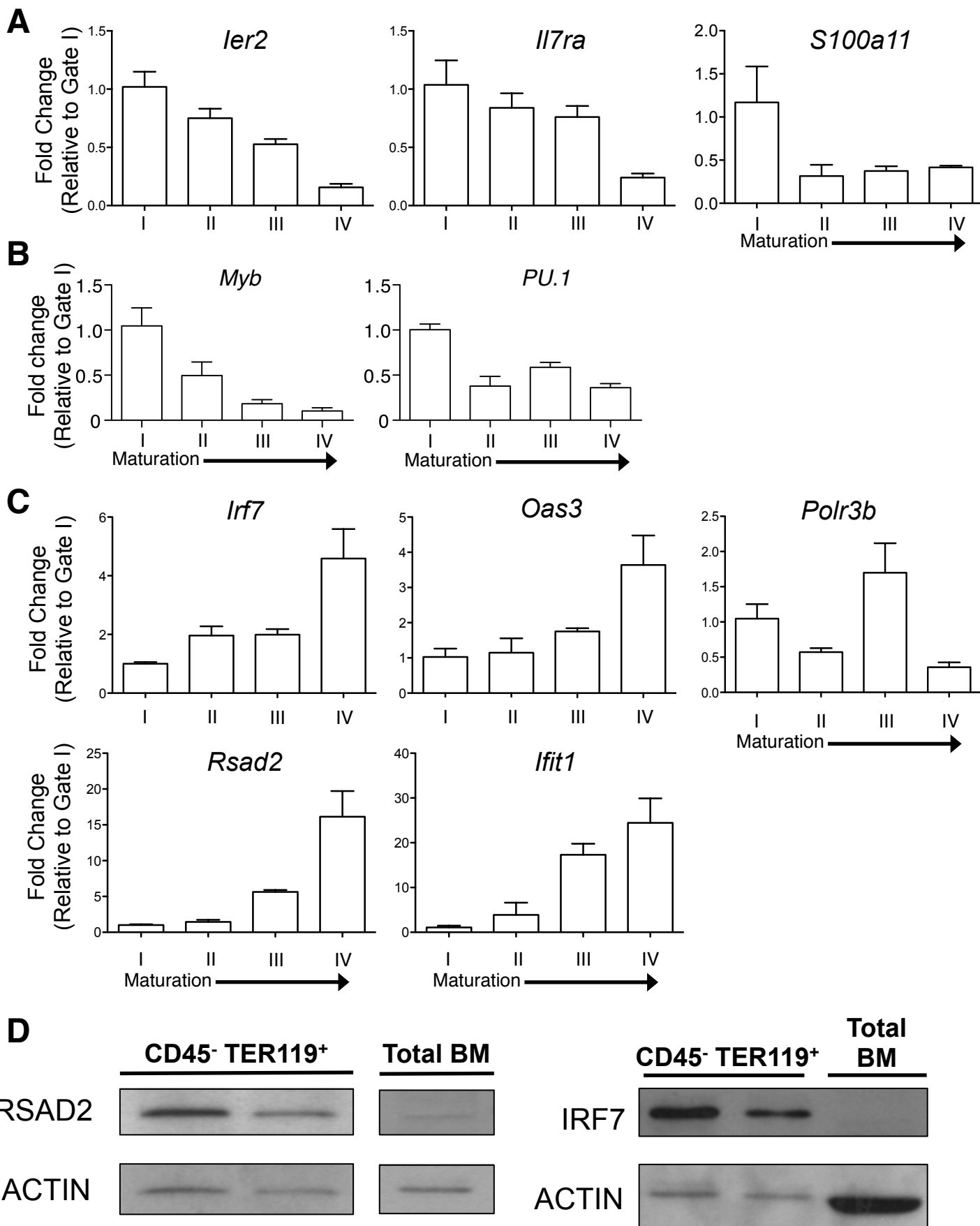

Supplement: S2 Fig — (A) qRT-PCR analysis of immune-related genes found to be downregulated over terminal erythroid maturation. Quantification of target genes is normalized to β actin and relative to expression within Gate I. (B) QRT-PCR gene expression analysis in WT bone marrow erythroblasts. (C) Validation of expression of immune-related genes found to be upregulated with erythroblast maturation in bone marrow CD45- Ter119+ fractions segregated by CD44 expression and FSC. Results are mean ± SEM of 3 cDNAs, each generated from one mouse. (D) Western blot expression analysis of IRF7 and RSAD2 in CD45-TER119+ FACS sorted bone marrow cells (n = 2 mice) as compared to total bone marrow (BM) cells (from right lane mouse). (PDF) [file pgen.1005526.s002.pdf]

**Figure S3****A**

|                                                      | Gate I | Gate II | Gate III |
|------------------------------------------------------|--------|---------|----------|
| Foxo3 expression (FPKM)                              | 78.1   | 102.6   | 156.5    |
| Differences between WT & <i>Foxo3</i> <sup>-/-</sup> | 1147   | 1010    | 3074     |

**B**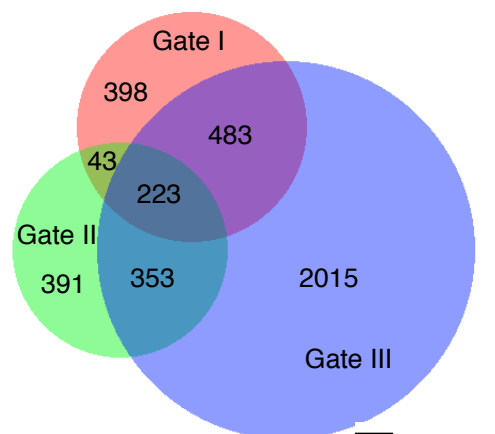**C**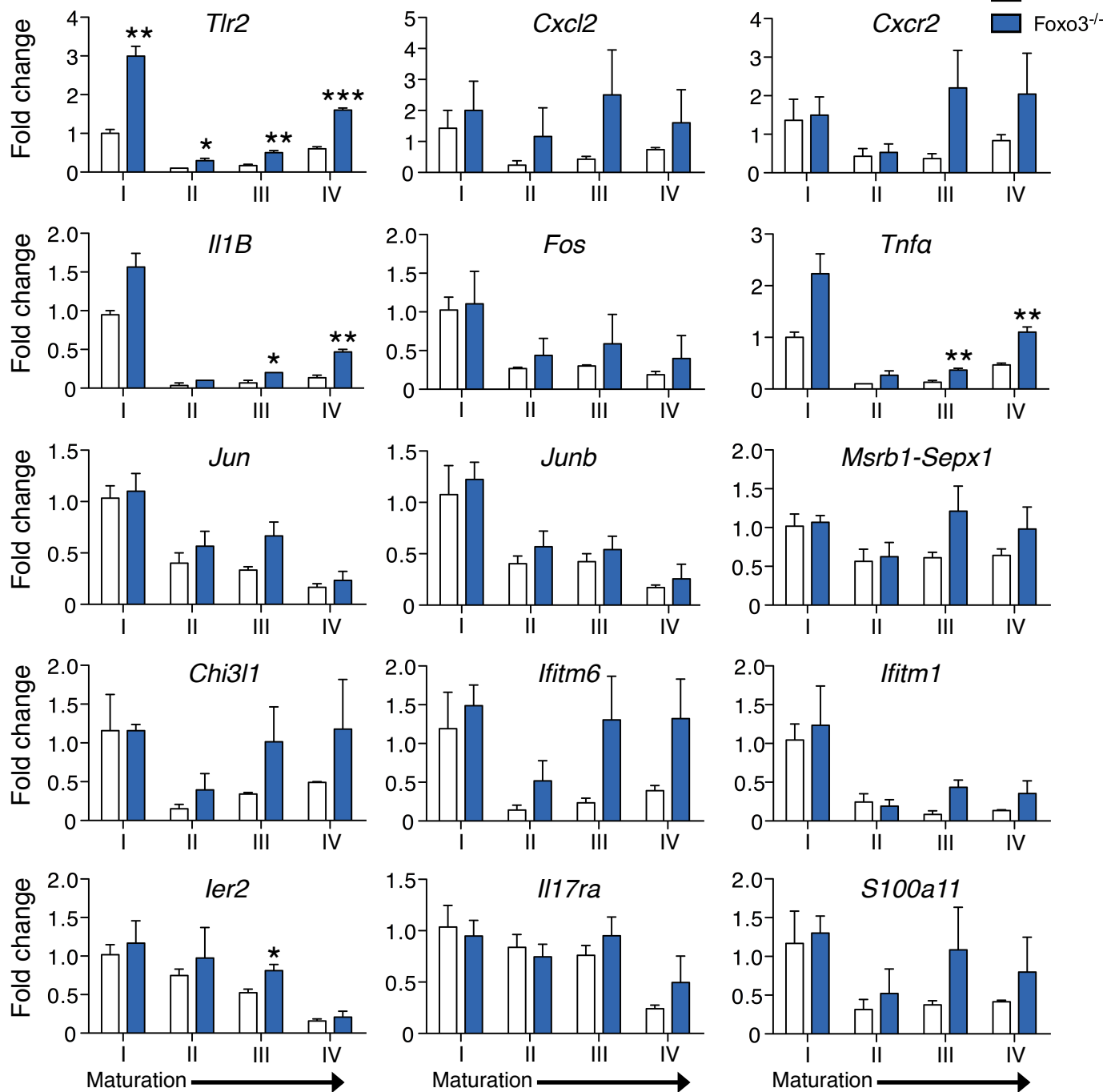

Supplement: S3 Fig — (A) The number of differentially expressed genes between WT and Foxo3 -/- erythroblasts at each gate during terminal erythroid maturation is shown together with the expression of Foxo3 in that particular Gate. (B) Venn diagram showing the overlap between the genes differentially expressed at each gate between WT and Foxo3 -/- erythroblasts. In total, 3904 distinct genes are differentially expressed. (C) QRT-PCR expression analysis of several immune-related genes differentially expressed between WT and Foxo3 -/- bone marrow Gates I to IV erythroblasts grouped in cluster J in Fig 1C. Expression data for Ier2, Il17rα, and S100a11 are from the same experiment in S2A Fig, with the addition of data from Foxo3 -/- erythroblasts. Quantification of target genes is relative to β actin. Results are mean ± SEM of 3 cDNAs, each generated from one mouse. *P < 0.05; Student’s t test. (PDF) [file pgen.1005526.s003.pdf]

Figure S4

A

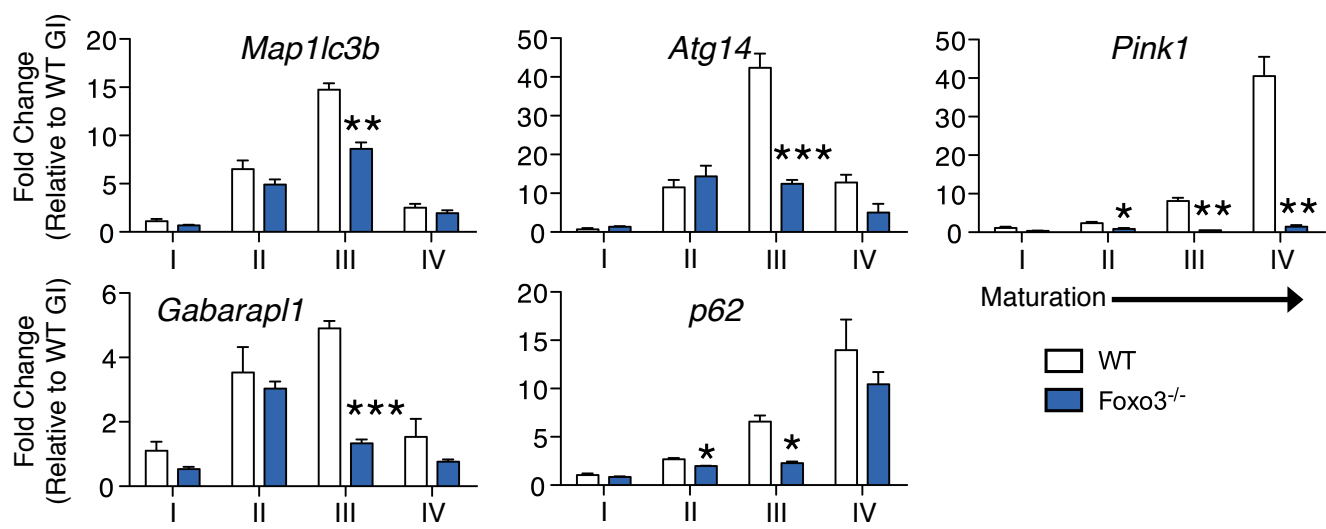

B

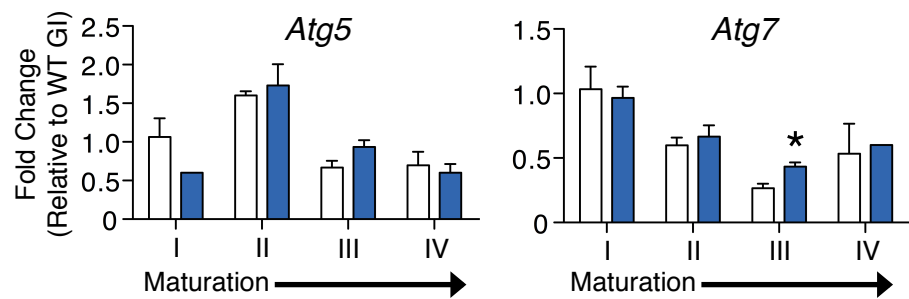

C

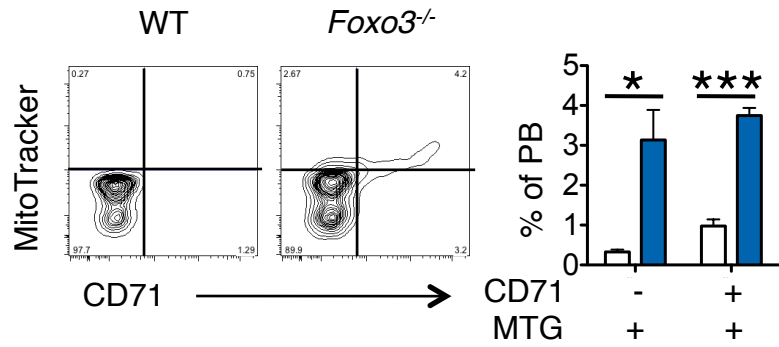

Supplement: S4 Fig — (A-B) QRT-PCR expression analysis of autophagy genes (A) including core autophagy genes (B) in WT and Foxo3 -/- Gate I to Gate IV erythroblasts. Quantification of target genes is normalized to β actin and relative to WT Gate I erythroblasts. Results are mean ± SEM of 3 cDNAs, each generated from one mouse. *P < 0.05; Student’s t test. (C) Flow cytometry analysis (left panels) and quantification (right panel, n = 4 in each genotype) of Mitotracker® Green in combination with CD71 surface expression of WT and Foxo3 -/- peripheral blood. *P < 0.05 **P < 0.01 ***P < 0.001, Student’s t test. (PDF) [file pgen.1005526.s004.pdf]

Figure S5

**A**

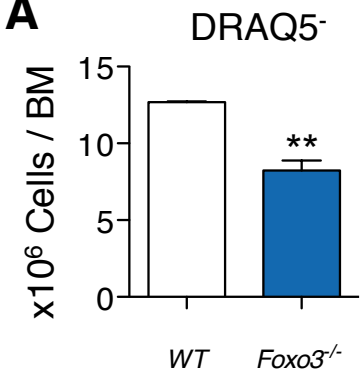

**B**

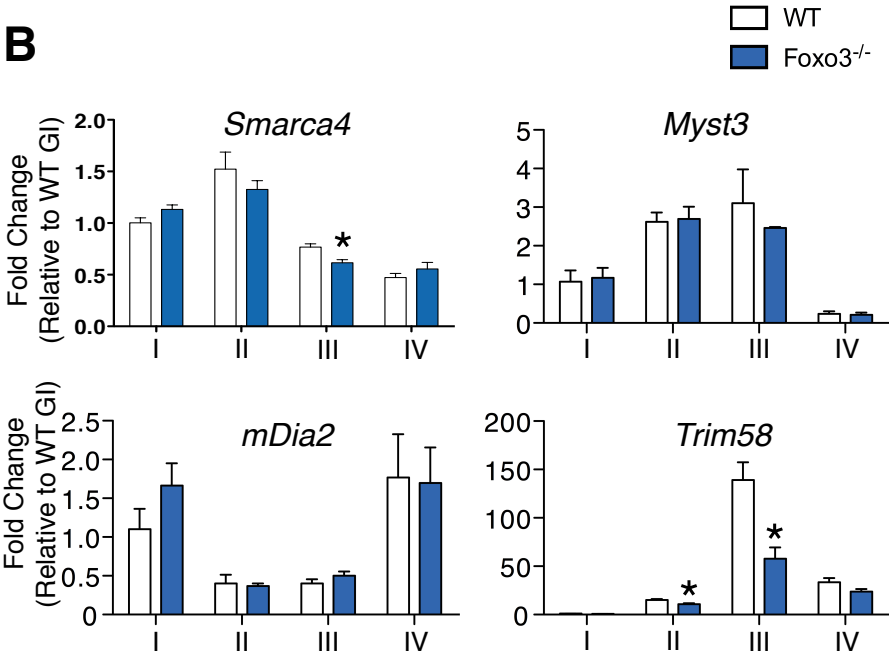

**C**

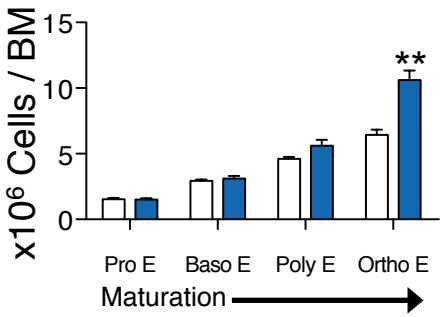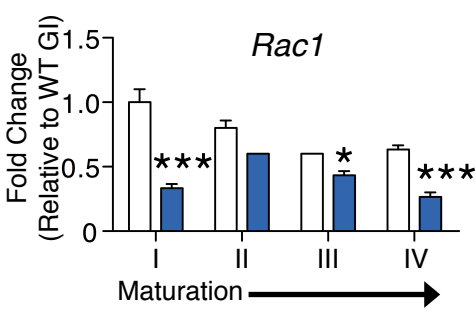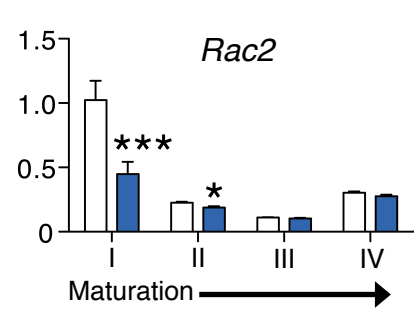

Supplement: S5 Fig — (A) Quantification of total number of WT and Foxo3 -/- bone marrow TER119+ DRAQ5- cells. Results are mean ± SEM of BM cells from three mice per genotype. (B) QRT-PCR expression analysis of genes implicated in chromatin condensation and enucleation in WT and Foxo3 -/- bone marrow Gates I to IV erythroblasts. Quantification of target genes is normalized to β actin. Results are mean ± SEM of 3 cDNAs, each generated from one mouse. (C) Quantification of total numbers of bone marrow WT and Foxo3 -/- pro, basophilic, polychromatic, and orthochromatic erythroblasts (from two femurs and tibias). Results are mean ± SEM of 4 mice per genotype. *P < 0.05, **P < 0.01, ***P < 0.001; Student’s t test. (PDF) [file pgen.1005526.s005.pdf]

Figure S6

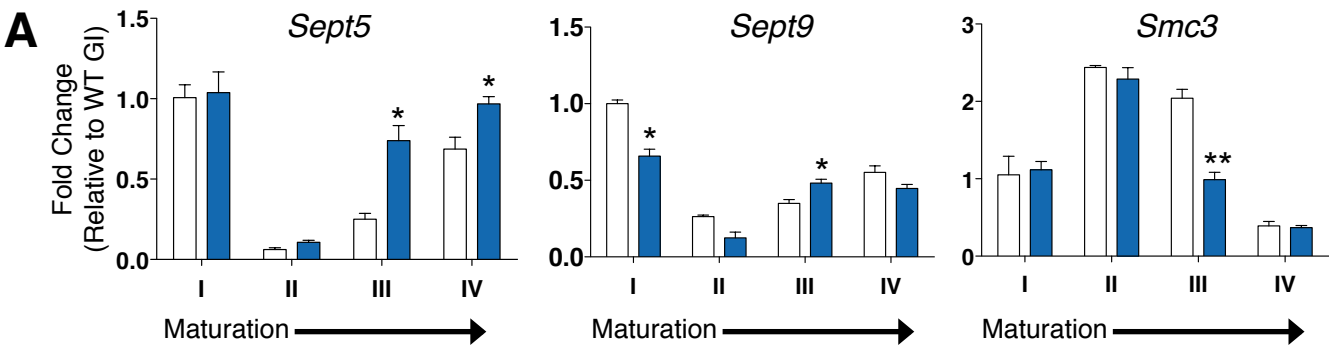

Supplement: S6 Fig — (A) QRT-PCR expression analysis of genes implicated in cytokinesis from FACS sorted WT and Foxo3 -/- erythroblasts from Gates I to IV. Quantification of target genes are normalized to β actin and relative to either WT Gate I. Results represent mean ± SEM of 3 cDNAs, each generated from one mouse. *P < 0.05, **P < 0.01; Student’s t test. ND; not done. (PDF) [file pgen.1005526.s006.pdf]

**Figure S7**

**A**

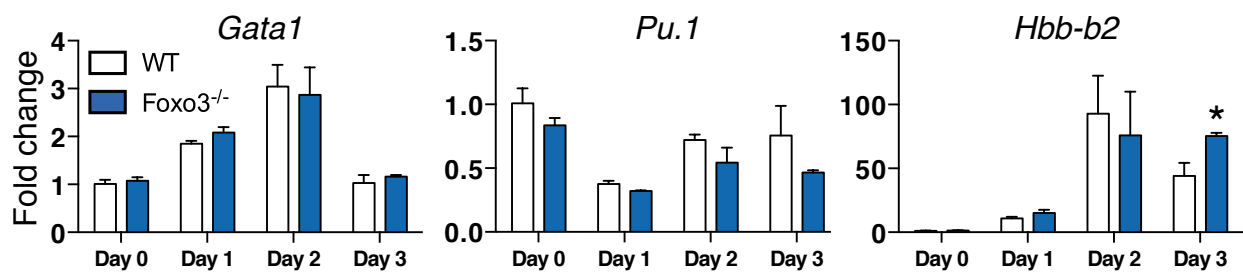

**B**

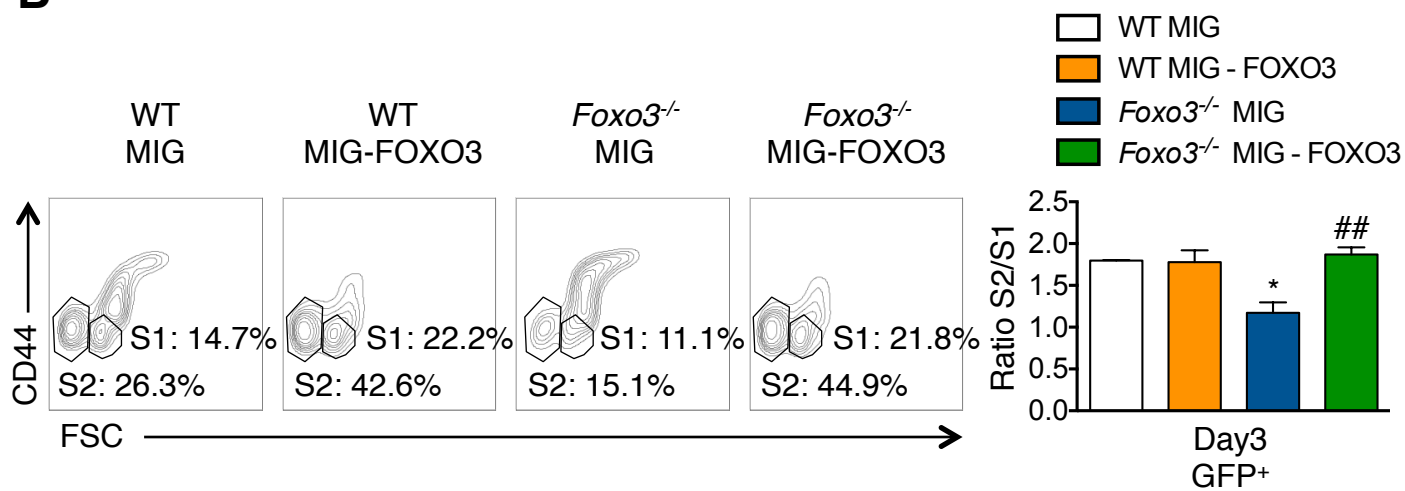

**C**

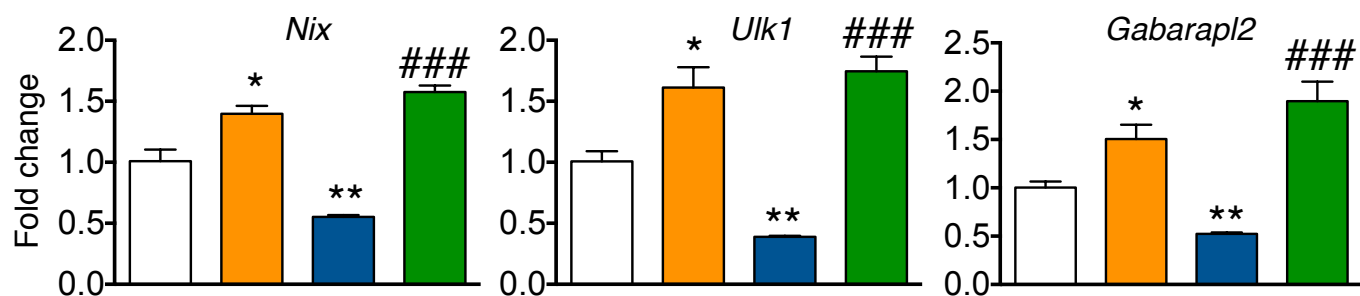

Supplement: S7 Fig — (A) QRT-PCR validation of erythroid gene expression after three days of maturation. WT and Foxo3 -/- BM cells were extracted and subjected to erythroid maturation. At least 105 cells were collected at each day and used to generate cDNA. Quantification of target genes is normalized to β actin and relative to WT erythroblasts at Day 0. Results represent mean ± SEM, n = 3. *P < 0.05, **P < 0.01; Student’s t test. (B) Representative FACS plots of GFP+, TER119+ maturing erythroblasts from Fig 7, with gates S1 and S2, which segregate the P3 population into more (S2) and less (S1) mature populations (left panels). Ratio of the S2 to S1 frequencies (right Panel). Results represent mean ± SEM, n = 3. *P < 0.05 compared to MIG-transduced WT cells, ## P < 0.05 compared to MIG-transduced Foxo3 -/- cells; Student’s t test. (C) QRT-PCR expression analysis of autophagy genes from cultured erythroblasts transduced with empty vector (MIG) or MIG-FOXO3 at day 3 of maturation is shown. GFP positive cells were FACS sorted. Quantification of target genes is normalized to β actin and relative to WT erythroblasts at day 0. Results represent mean ± SEM, n = 3. *P < 0.05 compared to MIG-transduced WT cells, # P < 0.05 compared to MIG-transduced Foxo3 -/- cells; Student’s t test. (PDF) [file pgen.1005526.s007.pdf]

Figure S8

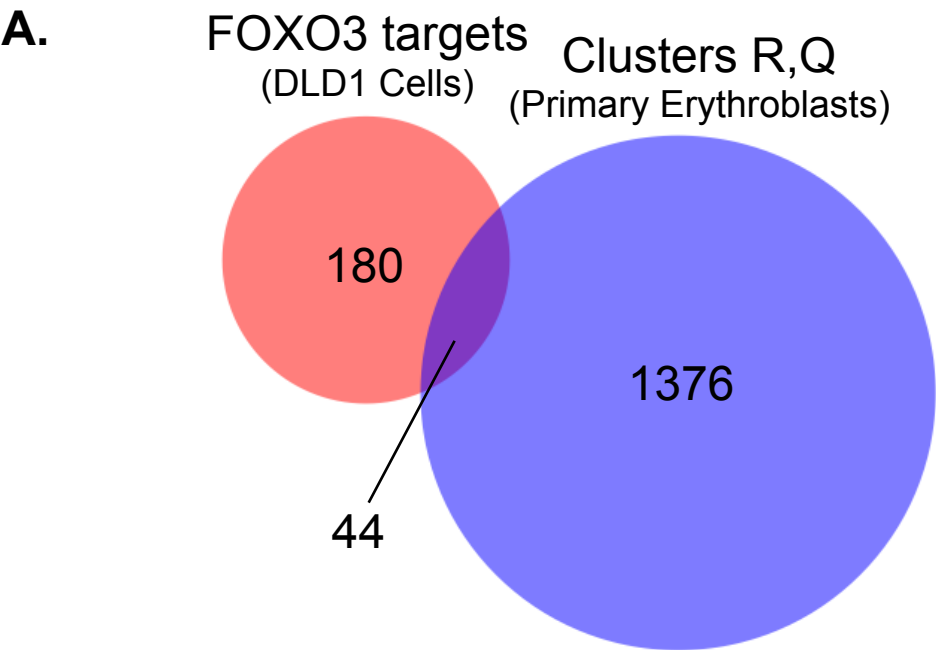

| Chi-Squared          | Observed | Expected |
|----------------------|----------|----------|
| ChIPseq - Overlap    | 44       | 14       |
| ChIPseq - No Overlap | 180      | 210      |

Supplement: S8 Fig — A list of genes considered to be directly activated by FOXO3 based on ChIP-seq peaks and RNA polymerase II occupancy from [77] (Red) of human DLD1 colon adenocarcinoma cell line was compared with genes from cluster Q and R (Blue). The data is displayed as a Venn diagram with overlapping gene shown in purple. A Chi squared test was performed to determine significance, with expected values based on an estimated number of total genes in the mouse genome as 23000. Chi squared equals 68.571 and the two-tailed P value is less than 0.0001. (PDF) [file pgen.1005526.s008.pdf]
